# Flow Reactor Study of NH_3_/DEE Oxidation Chemistry

**DOI:** 10.1021/acs.energyfuels.5c01874

**Published:** 2025-08-18

**Authors:** A. Ruiz-Gutiérrez, A. Bello-Gallego, M. U. Alzueta

**Affiliations:** Aragón Institute of Engineering Research (I3A), Department of Chemical and Environmental Engineering, 16765 University of Zaragoza, 50018 Zaragoza, Spain

## Abstract

The pursuit of environmentally sustainable alternatives to conventional fuels is essential today. Ammonia emerges as a promising candidate, though its inherent disadvantages must be addressed. Co-firing ammonia with fuels exhibiting superior thermochemical properties is one of the most widely accepted solutions. The present study investigates the oxidation of ammonia and diethyl ether mixtures (NH_3_/DEE). To this end, a quartz flow reactor was employed at atmospheric pressure, within a temperature range of 875–1425 K. The oxygen excess ratio (λ) and the NH_3_/DEE mixture ratio were systematically varied during the experiments. The results show an increase in the reactivity of ammonia when mixed with DEE. During pyrolysis, DEE is inhibited by competition for H radicals, which are essential for initial conversion steps. DEE undergoes thermal decomposition without a significant radical pool, while NH_3_ reactivity remains low. With the increase of the oxygen excess ratio, fuel oxidation occurs at lower temperatures. Hydroxyl (OH) and atomic oxygen (O) radicals are crucial in NH_3_ oxidation, while the presence of DEE derivatives further promote this oxidation, although both fuels exhibit competitive behavior regarding radical consumption. Variations in λ do not cause a significant effect in the oxidation temperature of DEE, with OH radicals playing a central role in the minor differences observed. Hydrogen abstraction via interaction with H radicals is the most important consumption reaction of DEE, mainly occurring at the secondary carbon position. Conversely, the production and interaction of derivatives at other positions contribute to the most inhibitory reactions in DEE oxidation. The NH_3_/DEE ratio has a significant impact on ammonia oxidation, particularly under high DEE dilution conditions (NH_3_/DEE = 10). OH and H radicals drive oxidation, while an increased DEE concentration leads to CH_3_ radical formation, enhancing fuel consumption. A literature-based kinetic mechanism, modified in the present work, was employed to represent and interpret the current results accurately.

## Graphical abstract

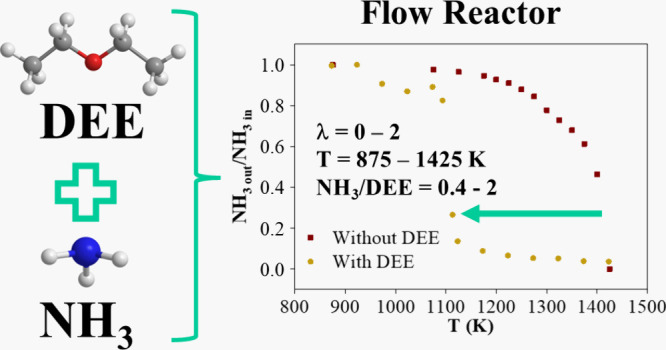

## 1 Introduction

Currently, one of the most challenging problems is the reduction of human impact on the environment. A major contributing factor is the emission of greenhouse gases into the atmosphere. The most emitted greenhouse gas is CO_2_. This compound is mostly produced after the combustion of conventional fuel. The combustion of carbon fuels is a major problem, since 80% of the energy produced comes from the combustion of those fuels.^1^ In the United States of America, approximately 33% of carbon dioxide emissions originate from fuel consumption in internal combustion engines of cars and trucks.^2^

In light of the foregoing, the Paris Agreement establishes an ambitious target to reduce net greenhouse gas emissions by 45% in 2030, making climate neutrality legally binding by 2050.^3^ To achieve reductions in CO_2_ emissions, incentives will be given to use renewable energies, consume less energy, reduce CO_2_ emissions from all means of transport and use more sustainable energy resources. Some of these alternative energy sources are hydrogen,^3^ ammonia^4^ or biofuels.^5^

Ammonia, being a carbon-free fuel, offers a promising avenue for reducing atmospheric CO_2_ emissions. At the same time, the technology for ammonia synthesis and storage is widely developed, with NH_3_ among the most synthesized products globally. The synthesis of this compound can also be carried out from renewable energy sources, such as solar, wind, or geothermal energy.^6^ Many of these technologies are proving competitive with conventional ammonia synthesis by the Haber–Bosch process.^7^ Ammonia derived from clean energies is called “green ammonia”.

However, using ammonia as a fuel does not only have advantages. For instance, its incomplete combustion can lead to NH_3_ emissions. Likewise, combustion with excess air may result in NO_*x*_ formation, which is released into the atmosphere. NO_*x*_ may further react within the atmosphere with organic compounds and ozone, promoting the formation of toxic species such as nitrate radicals, nitroarenes, and peroxides, thereby contributing to photochemical smog. They can also react with oxygen and water, causing acid rain.^8^ The incomplete combustion of ammonia also gives rise to nitrous oxide (N_2_O), a greenhouse gas approximately 300 times more harmful than CO_2_ and more long-lasting in the atmosphere. As a noncarbon compound, NH_3_ exhibits a low calorific value, reduced flame velocity, limited flammability, a high self-ignition temperature, and a high explosion limit.^9,10^

To address these challenges, cofiring ammonia with other fuels demonstrating superior thermodynamic properties may be a viable solution. Adding oxygenated organic compounds could be an alternative to the problem presented, since oxygenated compounds contain oxygen atoms in their molecular structure and have been used as fuel additives since the 1970s.^11^ The addition of these compounds, even in minimal concentrations, has been shown to reduce soot emissions in diesel fuels, enhance the antiknock properties of gasoline, and improve combustion efficiency.^12,13^

The investigation of simple oxygenated compounds such as dimethyl ether (DME) and methanol (CH_3_OH) has been extensively explored. Research has demonstrated that the addition of these fuels enhances the conversion of ammonia,^14,15^ shortens the ignition delay times (IDTs),^16,17^ and increases laminar burning velocities (LBVs).^18–20^ The incorporation of these additives promotes more stable and complete ammonia oxidation at lower temperatures.

Building on the positive outcomes achieved with both oxygenated compounds, it is considered necessary to investigate other, somewhat more complex compounds, which have also been previously reported as fuel additives. In this context, diethyl ether (DEE) and dimethoxymethane (DMM) represent the logical progression of the study. Both have been considerably less studied due to their complexity and the addition of a major carbon concentration to the mixture. Nevertheless, both mixtures have been documented to result in promising results when combined with ammonia.^21–23^

DEE is generally used as a solvent and contributes to various chemical synthesis processes. However, it has also been used as an additive to diesel,^24^ due to its favorable thermochemical properties, such as its high oxygen content, high cetane number, low autoignition temperature, and low cloud and pour points. As an additive, DEE can be used in diesel engines without system modifications.^25^ This compound improves both the viscosity and the cold flow properties of diesel fuel.^26^ In addition, global emissions are reduced due to the high cetane number. Its elevated oxygen content may help to oxidize ammonia, and even to reduce NO_*x*_ emissions under specific operating conditions.^27^

DEE exhibits superior atomization performance compared to diesel fuel due to its high Reynolds number and low Ohnesorge number.^28^ Moreover, with only 5% DEE addition, the properties of the mixture are optimal, enhancing engine performance.^29,30^ At the same time, soot production is reduced because the increase in oxygen, along with the lower content of aromatic compounds, contributes to the reduction of soot formation and, if formed, increases the reactivity of soot.^31^ However, DEE also has some disadvantages when it is used in engines. Chauhan et al.^27^ noted that a high percentage of DEE can cause problems such as cavitation in engines, due to a decrease in lubrication.

Although the use of DEE as an additive to diesel is the most studied mixture, DEE has also been added to different types of biodiesel fuels.^32,33^ Venu et al.^33^ compared the use of different additives (DEE, methanol, and ethanol) mixed with biodiesel. The study found that DEE is the additive that exhibits the most favorable results, increasing performance with minimal emissions.

For NH_3_/DEE mixtures, DEE has been used to improve ammonia combustion. In this regard, shock tube,^22,34^ rapid compression machine (RCM),^23^ and constant volume spherical vessel (CVSV)^35^ devices have been used. With the addition of small amounts of DEE, good results have been achieved for NH_3_ combustion. In shock tubes, it has been shown that 5% DEE reduces the ignition delay time by 80% compared to the same conditions for net NH_3_. Similar results are obtained for RCM, with 10% DEE, and similar ignition delay times to those observed for FACE (fuel for advanced combustion engines) F gasolines were achieved. Another important combustion parameter is the laminar flame speed, which is favored by adding DEE to NH_3_, improving as the proportion of DEE in the fuel mixture increases. The explosion limit of ammonia is also greatly enhanced with the addition of 0.5% DEE.^10^

Results by Garcia-Ruiz et al.,^36^ addressing the conversion of NH_3_/DEE mixtures at high pressure, indicates that increasing that pressure accelerates fuel consumption, with DEE being particularly favored.

Since the NH_3_/DEE fuel mixture has demonstrated promising results, further research is crucial for a deeper understanding of the conversion mechanism within this mixture. While some studies have been conducted, detailed species profiles for the most relevant species formed during fuel oxidation remain insufficient. In this context, the present work addresses the conversion of NH_3_/DEE mixtures from both experimental and kinetic modeling points of view. In particular, this study will explore a broader temperature range (875–1425 K) than previous studies, along with a selection of operating conditions, such as the NH_3_/DEE mixture ratio (0.4–10.4) and the oxygen excess ratio (λ) (0.5–2). The experimental results of the present work are simulated with a literature mechanism updated to account for the new experimental results, as described later.

## 2 Experimental Methodology

The experiments are conducted using a quartz flow reactor system. The dimensions of the reactor are 8.7 mm in diameter and 200 mm in length. An electrically heated oven is used to reach isothermal conditions in the reactor. To this end, a precision of ±5 K was achieved in the temperature profile for each measurement. Temperature quantifications are taken with a type-K fine-wire thermocouple. All the temperature profiles along the reaction zone (20 cm) are illustrated in Figure S1 of the Supporting Information.

Mass flow controllers (Alicat) are used to control the flows. A total gas flow rate of 1000 mL·min^–1^ (STP) was maintained throughout the experiments, resulting in a gas residence time of 190 ms at 1000 K, calculated based on temperature and pressure.

All the reactive gases are diluted in argon. Reagents are sourced from gas cylinders supplied by NIPPON Gases. All gases are introduced through separate injectors. Additionally, the primary gas flow (argon) was preheated prior to mixing with the other gases. A comprehensive description of the experimental setup can be found in the setup diagram provided in Figure 2 of the Supporting Information. Additionally, a more detailed reactor diagram is included in Figure S3 of the Supporting Information.

To quantify the outlet gases, an Agilent 990 micro GC gas chromatograph, equipped with thermal conductivity detectors (TCD), has been used to measure DEE, CH_3_CHO, C_2_H_5_OH, CO, CO_2_, CH_4_, C_2_H_6_, C_2_H_4_, C_2_H_2_, NH_3_, N_2_O, H_2_, N_2_, O_2_, and HCN. The error was calculated from the standard deviation and remained constant across the temperature range. It is an estimation of the experimental micro GC error associated with the oxidation of the mixture of fuels. The largest uncertainty associated with the measurements for each temperature is ± 10 ppm. Since the micro GC does not provide reliable measurements about NO, the outlet gas stream was also analyzed using an Advance Optima AO2020 series continuous gas analyzer. The NO analyzer has a measurement uncertainty of 1%, with a minimum detection limit of 10 ppm. NO_2_ concentrations are negligible in all the conditions studied. To cover the broadest range of conditions possible, temperature, oxygen excess ratio (λ), and NH_3_/DEE ratio were regulated for each experiment. The specific conditions for each experimental set are presented in Table 1.

**Table 1:** Experimental Conditions^a^

set	NH_3_	DEE	NH_3_/DEE	λ	*t*_r_ (s)
1	256	512	0.50	0	189/*T* (K)
2	456	507	0.90	0	191/*T* (K)
3	476	514	0.93	0.51	191/*T* (K)
4	467	470	1.00	0.98	190/*T* (K)
4R	476	490	0.97	1.02	188/*T* (K)
5	475	514	0.92	1.90	191/*T* (K)
6	1976	190	10.42	0.50	189/*T* (K)
7	2000	191	10.44	1.00	190/*T* (K)
8	981	96	10.22	1.90	191/*T* (K)
9	228	484	0.47	0.51	190/*T* (K)
10	231	487	0.47	1.02	189/*T* (K)
11	200	485	0.41	2.03	190/*T* (K)

a Balance is achieved with Ar. Concentrations are expressed in ppm.

λ is defined as the ratio of the initial oxygen concentration supplied during the experiment divided by the stoichiometric oxygen. The required concentrations were calculated based on reactions (r1 and r2).

r1 $${\mathrm{NH}}_{3}+0.75O_{2}\to 0.5N_{2}+1.5H_{2}O$$

r2 $${\left(C_{2}H_{5}\right)}_{2}O+6O_{2}\to 4{\mathrm{CO}}_{2}+5H_{2}O$$

Sets 1 and 2 in Table 1 are pyrolysis experiments for different NH_3_/DEE ratios to evaluate the influence of thermal decomposition and the influence of radicals provided by DEE. The impact of oxygen and NH_3_/DEE mixture ratio was studied together. Sets 3–5 in Table 1 show the influence of oxygen for the same concentration of both NH_3_ and DEE. Additionally, experiments 4 and 4R are repeated experiments. Finally, several experiments were conducted with different λ, always between 0.5 and 2, and all of them were carried out with NH_3_/DEE ratios in the range of 0.4–10.4. These experiments are represented in Table 1 as sets 6 to 11.

To evaluate the quality of experiments and determine if the main produced species are analyzed and quantified, mass balances were performed for each experimental set. The analyzed compounds from the experiments were used to conduct these balances. All balances are available in Figure S4 of the Supporting Information. Additionally, the same balances were performed using calculated data. These balances only show a margin of error of 10% relative to the initial amount of nitrogen and carbon added in high-reactivity regions, where precisely capturing all compounds becomes challenging. The discrepancies in the mass balances are more frequent for carbon and rarely occur for nitrogen. Similar trends are also observed in the calculated balances. A different behavior from the one described above is only observed in pyrolysis studies, which will be discussed in later sections.

## 3 Kinetic Mechanism

A mechanism proposed in our previous study on NH_3_/CH_3_OH mixtures has been used as a basis for the present work.^15^ This mechanism is primarily based on the Glarborg et al.^37^ mechanism. Alzueta et al.^38^ detailed some minor updates for NH_3_,^39^ CH_3_CN^40^ and NH_3_–NO^41^ subsets. Additionally, for the study on NH_3_/DME and NH_3_/DME/NO mixtures,^14^ reactions proposed by Marrodán et al.^12^ were added. Concerning NH_3_/CH_3_OH mixtures, reactions involving interactions between NH_2_–CH_3_OH radicals,^42,43^ new interactions between methanol derivatives and nitrogen compounds^44^ and their decomposition^45^ were incorporated, together with interactions between nitrogen and other carbon compounds.^46^ A more detailed explanation of the primary mechanism used can be found in Ruiz-Gutiérrez et al.^15^

The current mechanism incorporates the necessary reactions to simulate DEE oxidation, which has been taken from the mechanism of Tran et al.^47^ Some of the most important reactions in this model are hydrogen abstraction reactions, with the most significant being those proposed by Tran^47^ for H radicals, DEE + H ⇌ CH_3_CH_2_OCHCH_3_ + H_2_ (r3), Zhou et al.^48^ for OH radicals, DEE + OH ⇌ CH_3_CH_2_OCHCH_3_ + H_2_O (r4), and Yasunaga et al.^49^ with thermal decomposition of DEE, DEE (+M) ⇌ C_2_H_5_OH + C_2_H_4_ (+M) (r5). Another key addition to this mechanism is the oxidation reaction of DEE by oxygen, forming ethyl vinyl ether (EVE). The reactions describing this process are compiled from Sakai et al.^50^

Although the addition of the DEE subset from Tran et al.^47^ includes the main oxidation pathway for DEE, it lacks important interactions between nitrogen and carbon compounds. Thus, numerous interactions between DEE and nitrogenous compounds^51^ were incorporated by Shrestha et al.,^35^ as well as hydrogen abstraction from DEE by NH_2_ radicals,^52^ together with some interactions for the DEE, estimating these interactions to those with the DME^52^ have been included in the mechanism in the present work. Modifications of the DEE^53,54^ and ethylamine^55–57^ reaction subsets have also been added. A more detailed explanation of the implementation of these changes can be found in the original work of Shrestha et al.^35^

To verify the improvements introduced by the model updates, Figures S5.1 and S5.2 have been included in the Supporting Information. These Figures present the calculations performed using the NH_3_/DME and NH_3_/DME/NO mechanisms^14^ in combination with the Tran et al. mechanism,^47^ which is considered as the initial mechanism, as well as with the updated mechanism of the present work. The results indicate that the original model overestimates the consumption of the fuel mixture, whereas the updated model yields predictions that are more consistent with the experimental fuel consumption. Some of the most significant updates include the addition of C–N interactions proposed by Lucassen^56^ and Shrestha,^35^ as well as hydrogen abstraction from DEE by NH_2_ radicals.

Chemkin-Pro was employed to calculate the species profile behavior, using the plug flow reactor (PFR) module for the experimental configuration, with a constant temperature inside the reactor, since isothermal conditions can be assumed within ±5 K. All calculations were conducted using a standard flow rate of 1 L·min^–1^. The initial concentrations are those shown in Table 1. For selected conditions, sensitivity or reaction pathway analysis were performed using the Chemkin-Pro tool.

## 4 Results and Discussion

### 4.1 Pyrolysis

This study aims to determine the pyrolysis regime of NH_3_/DEE mixtures and to identify potential interactions between the fuels in the absence of oxygen within a flow reactor. Figure 1 shows the species profiles of the main identified compounds under pyrolysis conditions. Throughout this work, experimental data are represented by symbols, while calculated results are shown as lines. DEE undergoes thermal decomposition within the temperature range of 925–1075 K. The presence of NH_3_ impacts the conversion of DEE and inhibition of DEE consumption is observed when NH_3_/DEE mixture ratio is 1. The observed DEE consumption is primarily attributed to thermal decomposition, with DEE (+M) ⇌ C_2_H_5_OH + C_2_H_4_ (+M) (r5) as the reaction with the highest DEE consumption. At the same time, other reaction paths appear due to the presence of H and CH_3_ radicals, promoting hydrogen abstraction in reactions such as DEE + H ⇌ CH_3_CH_2_OCHCH_3_ + H_2_ (r3), and DEE + CH_3_ ⇌ CH_3_CH_2_OCHCH_3_ + CH_4_ (r6). A sensitivity analysis performed at 948 K (10% DEE conversion) further confirms that the previously mentioned reactions are the most influential in DEE oxidation. The sensitivity analysis can be found in Figure S6 of the Supporting Information (Figure S6.1 for DEE). However, interactions with CH_3_CH_2_OCH_2_CH_2_ and consumption of CH_3_ and H radicals by different pathways act to inhibit DEE oxidation.

**Figure 1:**
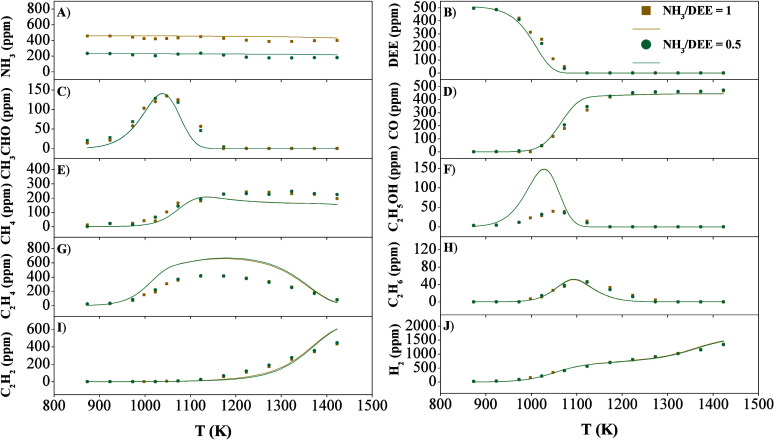
Concentrations of the key species produced under pyrolysis conditions for different NH_3_/DEE ratios (sets 1–2 of Table 1).

Two of the most important intermediate compounds in the pyrolysis of DEE are CH_3_CHO (Figure 1C), C_2_H_5_OH (Figure 1F), and C_2_H_4_ (Figure 1G). Although at low temperatures, the production of C_2_H_4_ and C_2_H_5_OH is mainly due to the thermal decomposition of DEE (r5), C_2_H_4_ is also formed by reactions such as C_2_H_5_ (+M) ⇌ C_2_H_4_ + H (+M) (−r7), and CH_3_CH_2_OCH_2_CH_2_ ⇌ C_2_H_5_O + C_2_H_4_ (r8). C_2_H_5_OH is formed within the temperature range 975–1175 K, exclusively produced through reaction (r5). The negative sign means that the reaction is occurring in the reverse sense as is written in the mechanism. Afterward, the remaining ethanol-consuming reactions involve both ethanol decomposition, C_2_H_5_OH (+M) ⇌ C_2_H_4_ + H_2_O (+M) (r9), and interactions with CH_3_ and H radicals, C_2_H_5_OH + H ⇌ CH_3_CHOH + H_2_ (r10) and C_2_H_5_OH + CH_3_ ⇌ CH_3_CHOH + CH_4_ (r11). The calculations for this compound exhibit the highest degree of inconsistency with the experimental data. This inconsistency may arise due to an overestimation in the formation of ethanol from DEE, as the experimental consumption of C_2_H_5_OH is more important than what is anticipated by the kinetic model.

The reaction that forms C_2_H_5_OH, almost exclusively, is DEE (+M) ⇌ C_2_H_5_OH + C_2_H_4_ (+M) (r5). The reaction rate of this important reaction has been studied in a number of works, e.g., refs 58 and 59 . For that reaction, we have the kinetic parameters suggested by Yasunaga et al.^60^ which are widely adopted in the literature, for example in DEE oxidation studies^47,61^ and fuel mixtures studies involving NH_3_/DEE.^35^ However, since this reaction appears to be so influential and the difference among determinations of this rate in the literature are important, we have evaluated the impact of varying the rate of (r5), dividing it by 10, according to few experimental determinations of this rate which suggest lower value for the reaction rate of (r5).^62,63^ An example of the comparison of the experimental and calculated results with k5 and 0.1*k5 are shown in Figure 2. It is seen that a lower rate for reaction (r5) than that from Yasunaga et al. results in a better agreement of experimental and calculated data, in particular for both DEE and C_2_H_5_OH concentrations. Therefore, the present results confirm the relevance of this reaction for the conversion of the mixtures considered, and it would be desirable to have available an improved determination of this reaction rate in a next future.

**Figure 2:**
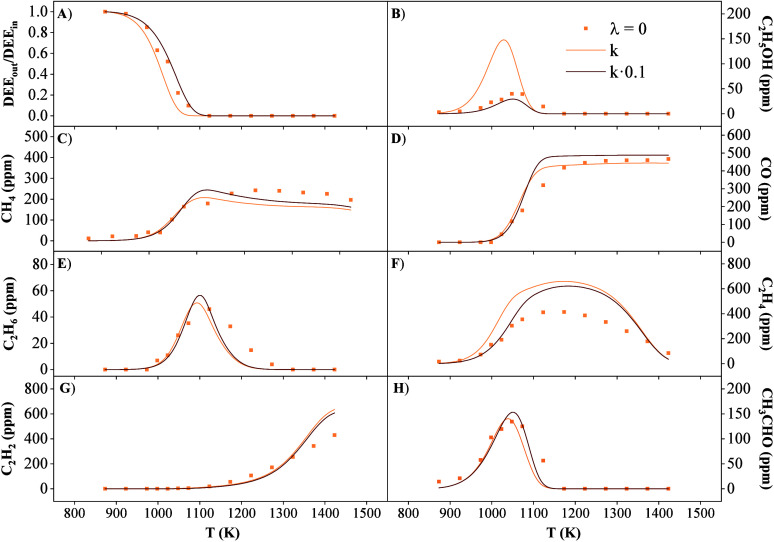
Comparison of experimental results and calculations made with the Yasunaga et al.^60^ rate for (r5), DEE (+M) ⇌ C_2_H_5_OH + C_2_H_4_ (+M), and the same rate divided by 10 for the pyrolysis conditions of set 1 in Table 1.

Methane (Figure 1E) and carbon dioxide (Figure 1D) are the final carbon-containing compounds formed during the pyrolysis of DEE. CH_4_ increases within the temperature range of 1000–1100 K, with the CH_3_ radical serving as the primary promoter of its formation. The most relevant reactions include C_2_H_4_ + CH_3_ ⇌ CH_4_ + C_2_H_3_ (r12) and CH_3_ + H (+M) ⇌ CH_4_ (+M) (r13). Carbon monoxide (CO) increases monotonically in the temperature range of 1025–1175 K, and it is produced through common reactions in DEE oxidation, CH_3_CO (+M) ⇌ CH_3_ + CO (+M) (r14) and HCO + O_2_ ⇌ CO + HO_2_ (r15). Both compounds, CH_4_ and CO, are reasonably well predicted by the model calculations.

Experimental carbon mass balances indicate a carbon lack of approximately 20% at high temperatures. This discrepancy is observed in both experiments (Sets 1–2) but is not reflected in the calculations. The experimental results can be explained by the formation of pyrolytic carbon, which was found at the end of these experiments. To our knowledge, no one has reported this behavior under these conditions for DEE or NH_3_/DEE mixtures. All the mass balances and an image of the material collected on the reactor walls are provided in the Supporting Information (Figures S4 and S7).

Ammonia does not show a different behavior for different DEE concentrations (Figure 1A). The sensitivity analysis carried out at high temperature (1425 K), Figure S6.2 in the Supporting Information, indicates that the reactions that most favor the oxidation of ammonia are NH_3_ + H ⇌ NH_2_ + H_2_ (r16) and NH_2_ + H ⇌ NH + H_2_ (r17). Simultaneously, the thermal decomposition of DEE (r5) plays a notable role in the oxidation of NH_3_. In contrast, the consumption of H radicals by other compounds, such as CH_3_ + H (+M) ⇌ CH_4_ (+M) (r13) and CH_3_CHO + H ⇌ CH_3_CO + H_2_ (r18), inhibit the potential oxidation of ammonia. The sensitivity analysis can be found in the Supporting Information, Figure S6 (Figure S6.2 for NH_3_).

### 4.2 Influence of the Oxygen Excess Ratio

Sets 1, 3, 4, and 5 in Table 1 were conducted to study the influence of oxygen in fuel oxidation. The species profiles for each compound are represented in Figure 3.

**Figure 3:**
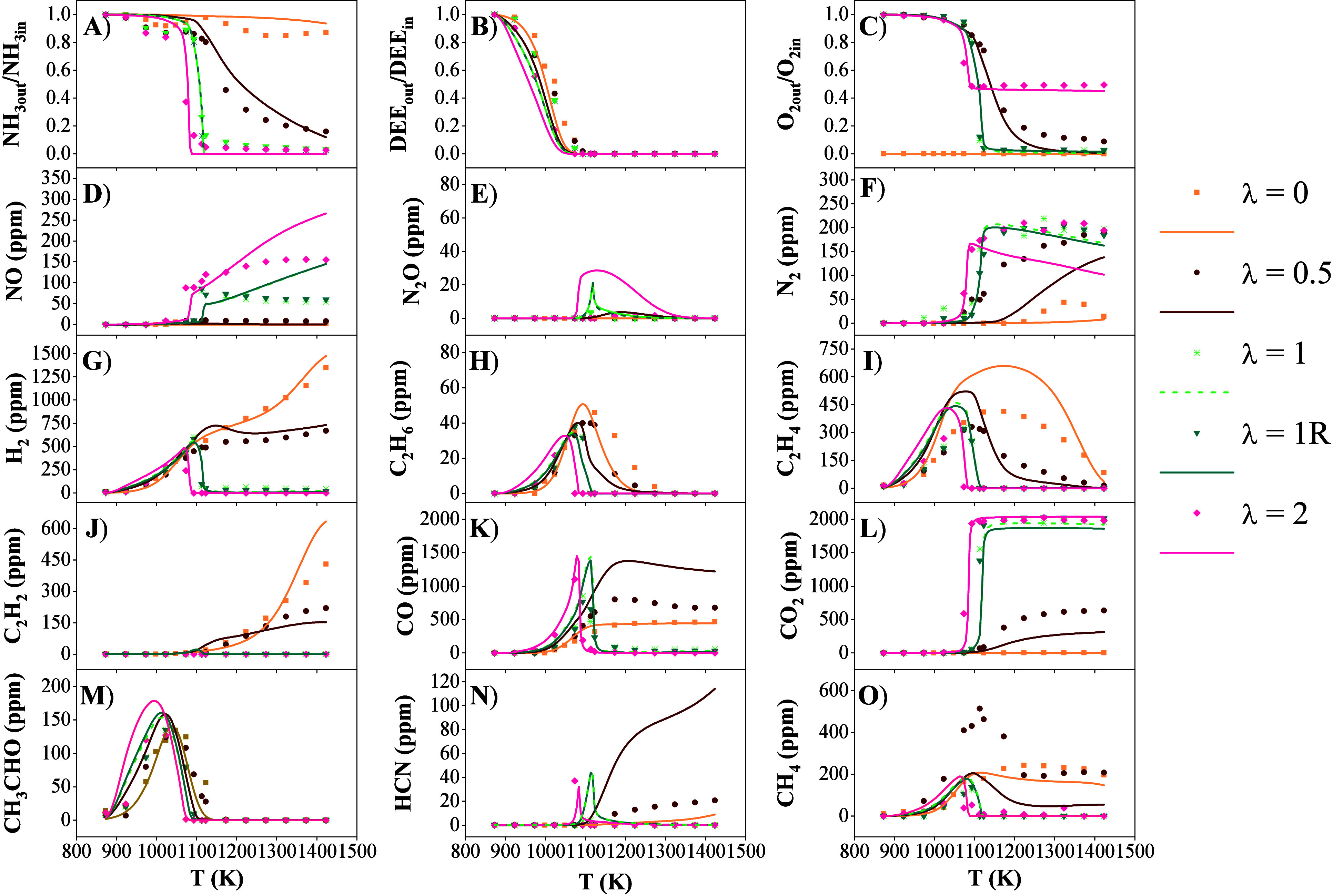
Concentrations of the principal product species for NH_3_/DEE = 1 for different λ (sets 1 and 3–5 of Table 1). NH_3_, DEE and O_2_, as reactants, are shown as normalized values.

Figure 3A shows the behavior of ammonia as a function of λ. It is observed that ammonia oxidation occurs at lower temperatures with higher O_2_ concentrations, with a temperature difference of 35 K between stoichiometric and fuel-lean conditions in the regions of higher reactivity. A similar behavior has been observed by García-Ruiz et al.^36^ Under fuel-rich conditions, NH_3_ is not completely consumed, with 16% of the ammonia remaining unreacted at higher temperatures. The calculated results show reasonable agreement with the experimental data.

The most important reactions in the oxidation of ammonia are NH_3_ + OH ⇌ NH_2_ + H_2_O (r19) and NH_3_ + O ⇌ NH_2_ + OH (r20), so the presence of OH and O radicals is key to favor the oxidation of NH_3_. Figure 4A shows the sensitivity analysis carried out for NH_3_, indicating that the reactions most favorable to the oxidation of ammonia produce OH radicals, with the most significant reactions being H + O_2_ ⇌ O + OH (r21), NH_2_ + HO_2_ ⇌ H_2_NO + OH (r22), and CH_3_ + HO_2_ ⇌ CH_3_O + OH (r23). This is similar to what has been observed in other studies,^22,35^ where (r21), driving the relative concentration of O, H and OH radicals, is the most influential reaction in NH_3_ oxidation.

**Figure 4:**
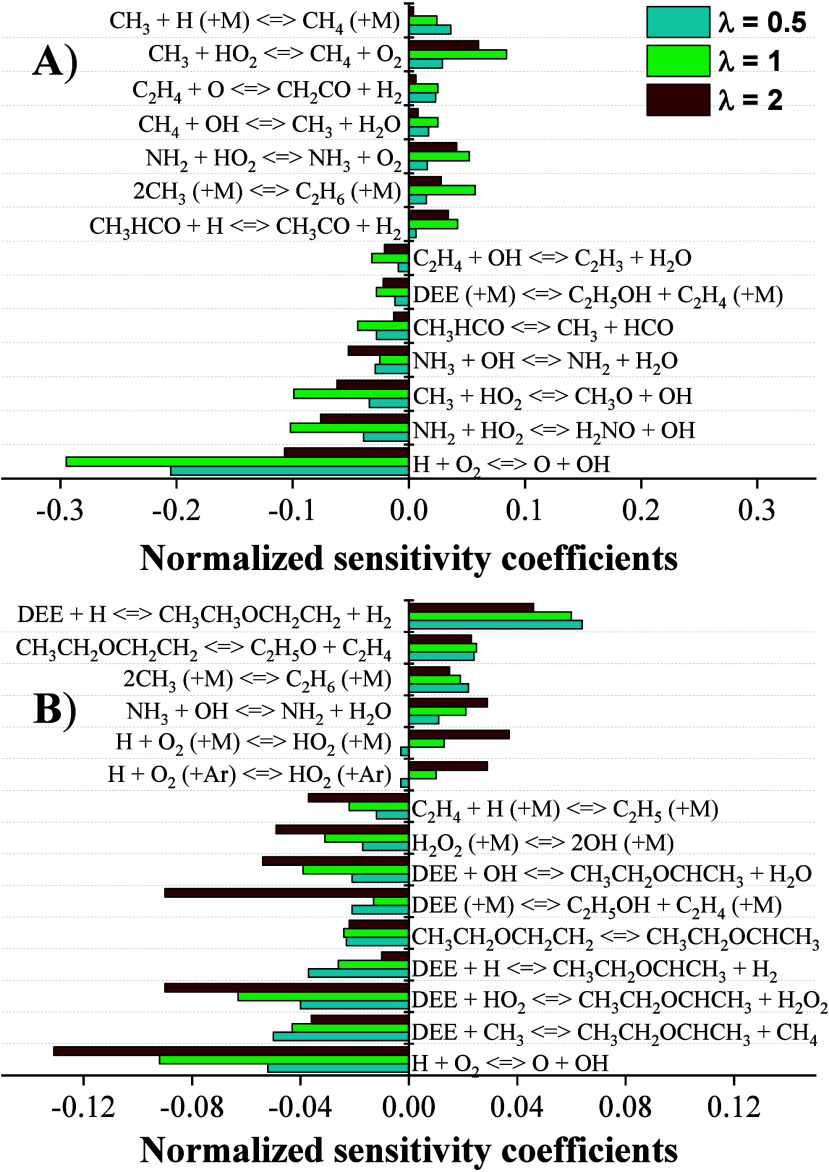
Sensitivity analysis for (A) NH_3_ and (B) DEE at 10% conversion for different λ. In ascending order of λ. Sets 3–5 in Table 1.

Additionally, the fact that reaction (r23) is identified as sensitive also indicates the beneficial role of DEE in promoting NH_3_ reactivity. In particular, it is worthwhile to mention that the decomposition of DEE (r5) does not require oxygen. At the same time, the generation of radicals from the decomposition of CH_3_CHO, CH_3_CHO ⇌ CH_3_ + HCO (r24), also results in increased reactivity of NH_3_. In contrast, the consumption of radicals, via pathways that do not involve interactions with nitrogen-containing compounds, hampers ammonia oxidation. Examples of this behavior include the occurrence of reactions CH_3_ + HO_2_ ⇌ CH_4_ + O_2_ (r25) or 2CH_3_ (+M) ⇌ C_2_H_6_ (+M) (r26), as chain terminating reactions.

Both H_2_ and C_2_H_4_, in NH_2_ + H_2_ ⇌ NH_3_ + H (−r27), and C_2_H_4_ + NH_2_ ⇌ C_2_H_3_ + NH_3_ (r28), are able to recycle back ammonia. Both H_2_ and C_2_H_4_ are directly produced mainly from the oxidation of DEE, so it can be concluded that the presence of DEE initially provides some inhibition to NH_3_ consumption. These reactions become less significant as the operating temperature increases.

The nitrogen compounds produced from the oxidation of NH_3_ are N_2_O, NO, N_2_, and HCN. Nitrous oxide (Figure 3E) has only been detected under fuel-lean and stoichiometric conditions, although the concentrations detected are negligible. The N_2_O detected under stoichiometric conditions corresponds to the temperature range where the highest concentration peak is observed in the calculations. This does not align with the comparison obtained under fuel-lean conditions.

NO (Figure 3D) is one of the most polluting emissions produced from the oxidation of NH_3_. The concentration of this pollutant increases with increasing λ. At the onset of NO production under stoichiometric conditions (1113 K), the significant role of carbon compounds in NO production is observed, where C_2_H_4_ + HNO ⇌ C_2_H_5_ + NO (−r29) occurs. On the other hand, once the temperature allows ammonia to become more reactive, (−r29) is no longer significant, and NO is produced by another pathway, through HNO: HNO + H ⇌ NO + H_2_ (r30), consistent with the results presented by Zheng et al.^22^ who also stated that the production of NO is primarily caused by (r30). For λ = 2, compounds such as C_2_H_4_ and C_2_H_5_ actively interact with nitrogen-containing compounds, producing NO. These interactions become important at high temperatures, as observed under stoichiometric conditions. At these elevated temperatures, the calculations overestimate the NO production, as was also observed in earlier studies addressing the conversion of this fuel mixture^23,35^ (Figure S8 of the Supporting Information).

Given the overestimation of NO at elevated temperatures, a sensitivity analysis was performed under stoichiometric conditions and at 1250 K, Figure S8 in the Supporting Information. It was observed that the interactions of O radicals with other radicals, such as NH or NCO are the most sensitive reactions. A comprehensive study of NCO + O ⇌ NO + CO (r31) and its reaction kinetic parameters would be desirable. It would also be interesting to have available an in-depth study of the consumption pathways of NH radicals, and related kinetic parameters of the involved reactions, since, according to Figure S8, reactions such as NH + H ⇌ N + H_2_ (r32) promote the non-formation of NO.

Regarding N_2_ (Figure 3F), its highest production as temperature increases is associated with the increased NH_3_ consumption. A difference of 150 K is observed between the onset of N_2_ production under fuel-rich and fuel-lean conditions. N_2_ production mainly happens through: NH_2_ + NO ⇌ N_2_ + H_2_O (r33) and NNH ⇌ N_2_ + H (r34).

HCN is a highly toxic compound (Figure 3N), with a maximum observed concentration of 40 ppm, in agreement with other studies at high pressure.^36^ Furthermore, in the presence of the necessary O_2_, the temperature range in which this secondary product is predominantly generated is very narrow. HCN production shifts to higher temperatures as the initial concentration of O_2_ is reduced, with HCNH + H ⇌ HCN + H_2_ (r35) being the reaction that contributes most to its production. Formation of HCNH arises from CH_3_NH_2_, which is the compound that also contributes to the generation of the majority of HCN concentration, according to Shrestha et al.^35^ Calculations show a significant discrepancy of HCN under fuel-rich conditions, with a notable overestimation in the performed calculations. According to the calculations carried out (1250 K), HCNH, CN, and CH_2_NH_2_ appear to be fundamental in the production of HCN. When these reactions were omitted, a notable discrepancy is observed, with a decrease of around 30 ppm, which suggesta slow consumption of HCN. Under the mentioned conditions, HCN consumption is driven by interactions with CH_3_, H, and O radicals.

H_2_ (Figure 3G) shows a significantly different behavior depending on λ, and shows to be present in a narrower temperature range relative to the amount of available H_2_. This is due to reactions that play a key role in H_2_ consumption, such as OH + H_2_ ⇌ H + H_2_O (r36) and NH_2_ + H_2_ ⇌ NH_3_ + H (−r27). Under fuel-rich conditions, these reactions are not significant, so the H_2_ concentration continues to increase at high temperatures. The production of H_2_ in all conditions studied results from hydrogen abstraction between DEE and H radicals, DEE + H ⇌ CH_3_CH_2_OCHCH_3_ + H_2_ (r3).

DEE (Figure 3B) reacts in a lower temperature range than NH_3_, with its reaction occurring in a temperature range of 925–1075 K. While DEE oxidation happens at lower temperatures as λ increases, the overall temperature range across all conditions remains quite narrow, with DEE being fully consumed in a 50 K difference. Another study confirms the low influence of λ on DEE consumption.^36^

Following the conditions studied at λ, it is shown that DEE is oxidized through its interaction with H and OH radicals. The hydrogen abstraction from DEE occurs predominantly from the second carbon position, with DEE + H ⇌ CH_3_CH_2_OCHCH_3_ + H_2_ (r3) and DEE + OH ⇌ CH_3_CH_2_OCHCH_3_ + H_2_O (r4) being the most important reactions in the oxidation of DEE. The hydrogen abstraction from the first carbon also occurs, with DEE + H ⇌ CH_3_CH_2_OCH_2_CH_2_ + H_2_ (r37) being one of the four most important consumption reactions under all conditions studied. The preference for carrying out hydrogen abstraction at this hydrogen atom location has been reported in other studies.^22,35,36^ As observed under pyrolysis conditions, the decomposition of DEE, DEE (+M) ⇌ C_2_H_5_OH + C_2_H_4_ (+M) (r5), becomes increasingly significant with decreasing λ. Therefore, it can be concluded that this pathway is of minor importance as long as there is a sufficient radical pool to facilitate the hydrogen abstraction of DEE.

Figure 4B shows a sensitivity analysis performed on DEE oxidation at the temperature corresponding to 10% of its consumption. It can be observed that many of the reactions that favor the oxidation of DEE are directly related to the production of OH radicals, such as H + O_2_ ⇌ O + OH (r21), and DEE + HO_2_ ⇌ CH_3_CH_2_OCHCH_3_ + H_2_O_2_ (r38). Furthermore, the sensitivity to reactions involving OH radicals increases, thereby enhancing their relevance as λ increases. This is due to the higher availability of radicals as the initial O_2_ concentration rises. At the same time, other DEE oxidation reactions, such as DEE + H ⇌ CH_3_CH_2_OCHCH_3_ + H_2_ (r3) and DEE + CH_3_ ⇌ CH_3_CH_2_OCHCH_3_ + CH_4_ (r6), become less significant as the concentrations of OH and HO_2_ radicals increase. Additionally, the hydrogen abstraction at the second carbon, facilitated by the migration of a H radical to this position, is favored under all studied conditions, resulting in CH_3_CH_2_OCH_2_CH_2_ ⇌ CH_3_CH_2_OCHCH_3_ (r39).

Concerning the reactions that most inhibit the oxidation of DEE, it is observed that the abstraction of an H from the first carbon when interacting with an H radical, DEE + H ⇌ CH_3_CH_2_OCH_2_CH_2_ + H_2_ (r37), is one of the most inhibitory reactions.^22^ This is because the reaction rate of (r37) is an order of magnitude lower than that of (r3). The consumption of OH radicals by NH_3_ also inhibits the oxidation of DEE, as both fuels compete for the consumption of the OH radical. In addition, the consumption of CH_3_ radicals for ethane production, 2CH_3_ (+M) ⇌ C_2_H_6_ (+M) (r26), also inhibits DEE oxidation, as (r26) is chain terminating and CH_3_ is an important radical for the initial hydrogen abstraction from DEE.

Acetaldehyde (CH_3_CHO; Figure 3M) is one of the most important intermediate compounds in the oxidation of DEE. This compound has been identified in the temperature range of 875–1123 K. The production of this compound shifts to higher temperature zones as λ decreases, coinciding with the reaction temperatures of DEE. The concentrations across the different cases studied do not differ significantly, with a discrepancy observed at λ = 2. The maximum peak of CH_3_CHO detected in these experiments is 134 ppm, corresponding to λ = 1. All the CH_3_CHO is produced via CH_3_CH_2_OCHCH_3_ ⇌ C_2_H_5_ + CH_3_CHO (r40). This observation was also reported by Shrestha et al.,^35^ who found that all products resulting from hydrogen abstraction of DEE undergo decomposition following a similar pathway to that of reaction (r40). In contrast, the consumption of CH_3_CHO is produced by its reaction with radicals H, OH, and CH_3_.

Other important products in the oxidation of DEE are C_2_H_6_ (Figure 3H), C_2_H_4_ (Figure 3I), and C_2_H_2_ (Figure 3J). All these compounds show increasing concentrations when λ decreases. C_2_H_6_ is formed via 2CH_3_ (+M) ⇌ C_2_H_6_ (+M) (r26), which is most favored at λ = 0.5. The C_2_H_4_ formed comes mainly from C_2_H_5_ (+M) ⇌ C_2_H_4_ + H (+M) (−r7), with C_2_H_5_ originating from the decomposition of CH_3_CH_2_OCHCH_3_, CH_3_CH_2_OCHCH_3_ ⇌ C_2_H_5+_ CH_3_CHO (r40). The ethylene concentration increases under fuel-rich conditions, highlighting the more important role of DEE (+M) ⇌ C_2_H_5_OH + C_2_H_4_ (+M) (r5) in these fuel-rich conditions. C_2_H_2_ is only found in fuel-rich conditions. This compound is produced from C_2_H_3_, in reactions C_2_H_3_ (+M) ⇌ C_2_H_2_ + H (+M) (−r41) and C_2_H_3_ + O_2_ ⇌ C_2_H_2_ + HO_2_ (r42). Although the formation of C_2_H_2_ is equivalent to that depicted by Shrestha et al.^35^ C_2_H_2_ has only been observed experimentally under fuel-rich conditions in this experimental setup. Additionally, C_2_H_3_ originates exclusively from C_2_H_4_ in reactions such as C_2_H_4_ + OH ⇌ C_2_H_3_ + H_2_O (r43) and C_2_H_4_ + NH_2_ ⇌ C_2_H_3_ + NH_3_ (r28). The relevance of the reaction (r28) indicates that the presence of C_2_H_4_ inhibits the oxidation of ammonia.

For CO (Figure 3K) and CO_2_ (Figure 3L), a behavior similar to that observed in previous studies of carbon-containing fuel mixtures is observed.^14,36,43^ For stoichiometric and fuel-lean conditions, CO formation only occurs in a very narrow zone, i.e. within a limited temperature range. The production of CO is caused by HCO oxidation, HCO + O_2_ ⇌ CO + HO_2_ (r44), and the decomposition of CH_3_CO, with CH_3_CO (+M) ⇌ CH_3_ + CO (+M) (r14). The consumption of CO coincides with the production of CO_2_, with the most important reaction being CO + OH ⇌ CO_2_ + H (r45), as usually reported in the literature for the fuel generation of CO_2_ during consumption of C compounds.^22^ The behavior of CO differs significantly under fuel-rich conditions, as no significant drop in CO concentration is found. Calculations show a discrepancy, overestimating CO production and, consequently, underestimating CO_2_ production. This may be due to the very high deceleration of the reactions involved in CO consumption, with an example of a deceleration of 4 orders of magnitude in the case of (r43). This behavior of the kinetic model has been observed in other studies.^23,35^

CH_4_ (Figure 3O) is formed over a wide temperature range (975–1425 K). The experiments under fuel-rich conditions do not show a total consumption of methane at any temperature, and in the case of pyrolysis, there is no prolonged consumption. This is because the main CH_4_ consumption reactions in this fuel mixture, CH_4_ + OH ⇌ CH_3_ + H_2_O (r46) and CH_4_ + O ⇌ CH_3_ + OH (r47), are of limited significance due to the high stability of the CH_4_ species and due to the lack of OH and O radicals. The CH_4_ production pathways vary depending on temperature, with reactions such as C_2_H_4_ + CH_3_ ⇌ C_2_H_3_ + CH_4_ (r48) and CH_3_ + H (+M) ⇌ CH_4_ (+M) (r13), which are relatively slow. These reactions become more important with higher temperature, where the hydrogen abstraction from DEE by methyl radicals, DEE + CH_3_ ⇌ CH_3_CH_2_OCHCH_3_ + CH_4_ (r6) and DEE + CH_3_ ⇌ CH_3_CH_2_OCH_2_CH_2_ + CH_4_ (r49), along with the consumption of CH_3_CHO, CH_3_CHO + CH_3_ ⇌ CH_3_CO + CH_4_ (r50), play a less important role due to the totally DEE consumption. Under fuel-lean conditions, the hydrogen abstraction of DEE becomes less significant for CH_4_ production due to the dominance of OH and O radicals in the hydrogen abstraction reaction of DEE.

### 4.3 Influence of the NH_3_/DEE Ratio

In this section, the behavior of the fuel mixture will be studied by varying the NH_3_/DEE mixture ratio (Figure 5). Emphasis will be placed on the compounds where significant changes in the calculated results were observed. Additionally, comparisons will be conducted at λ = 2 due to the better agreement between the experimental results and the calculations. The results of the comparisons for other λ conditions are provided in the Supporting Information, Figures S9 and S10.

**Figure 5:**
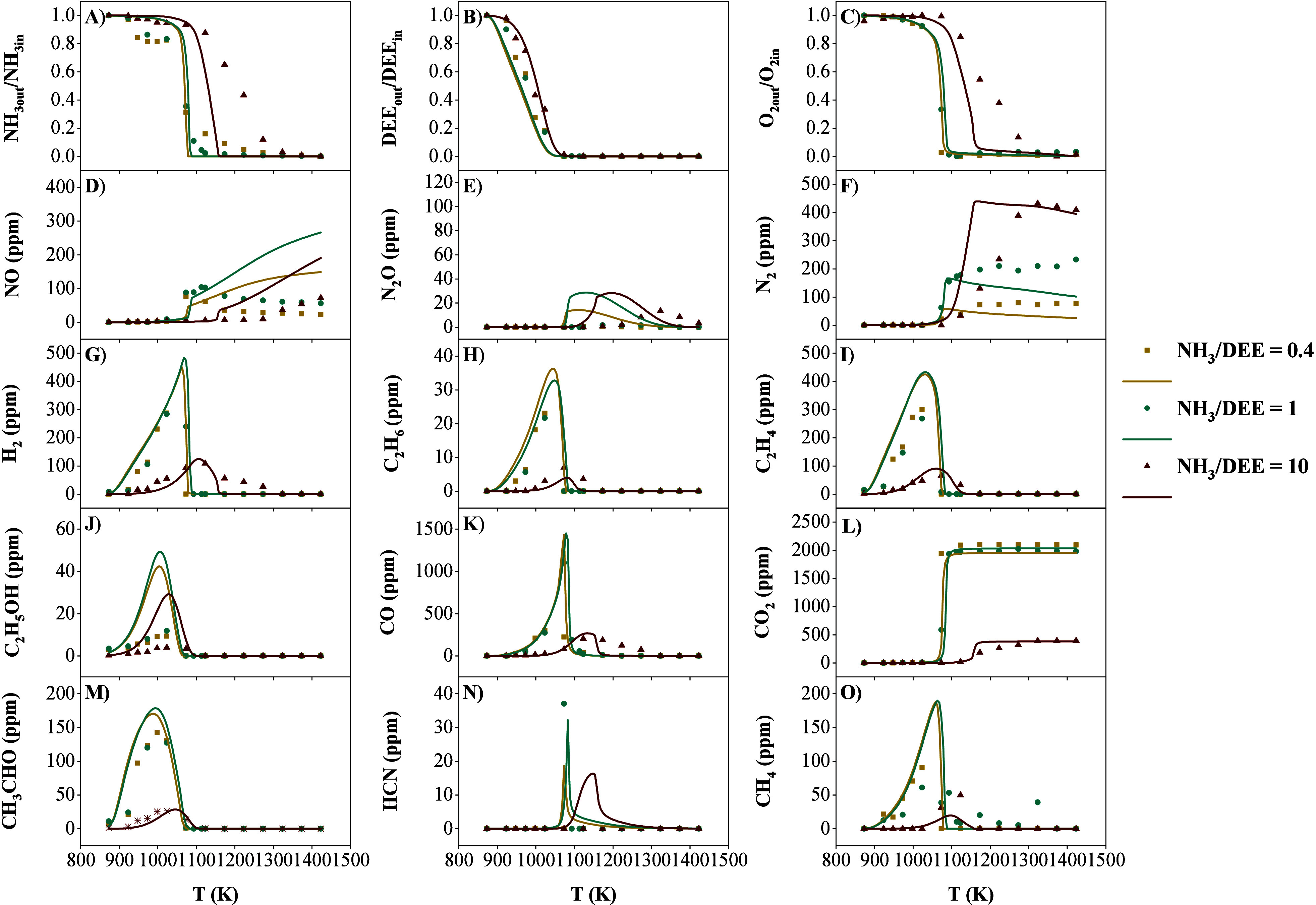
Concentrations of the most important product species for λ = 2 for different NH_3_/DEE ratios (sets 5, 8, and 11 of Table 1).

For NH_3_ (Figure 5A), it is noted that the conditions with the lowest oxidation temperature occur at NH_3_/DEE = 1, with a difference of 250 K compared to the most unfavorable condition (NH_3_/DEE = 10). Thus, having the same concentration of fuels favors the oxidation of NH_3_ at lower temperatures. For the case of NH_3_/DEE = 0.4, it is observed that from 1125 K, consumption of NH_3_ is reduced, resulting in proportionally higher concentrations than those obtained with equivalent fuel concentrations. It can be deduced from this that the addition of DEE results in a very high contribution to the radical pool^35^ and, therefore, on the conversion of NH_3_.

An increase in the ammonia oxidation temperature is observed for low DEE concentrations. This phenomenon may be attributed to a reduced significance of the primary NH_3_ oxidation reaction, NH_3_ + OH ⇌ NH_2_ + H_2_O (r19), thereby allowing secondary reactions, such as NH_3_ + O ⇌ NH_2_ + OH (r20), to become more prominent. This behavior is not observed for higher DEE concentrations, where ammonia is nearly entirely consumed through interaction with the OH radical (r19). This is because ammonia chemistry dominates in mixtures where DEE comprises less than 20%.^35^ Remarkably, the model overestimates NH_3_ consumption at NH_3_/DEE = 10, while providing a reasonable fit for the investigated cases.

Figure 6A presents a sensitivity analysis of NH_3_ when 10% of the fuel has been consumed. The OH radical production is crucial for NH_3_ consumption, with H + O_2_ ⇌ O + OH (r21) and NH_2_ + HO_2_ ⇌ H_2_NO + OH (r22) being among the most significant reactions for all mixture ratios. Also, the role of CH_3_ radicals in generating additional OH radicals, particularly through CH_3_ + HO_2_ ⇌ CH_3_O + OH (r23), which becomes increasingly significant as the proportion of DEE in the mixture rises. HO_2_ increases as the concentration of DEE increases, and CH_3_ radicals originate from the decomposition of a DEE intermediate compound, CH_3_CO (+M) ⇌ CH_3_ + CO (+M) (r14), highlighting the role of DEE in initiating the NH_3_ reaction.

**Figure 6:**
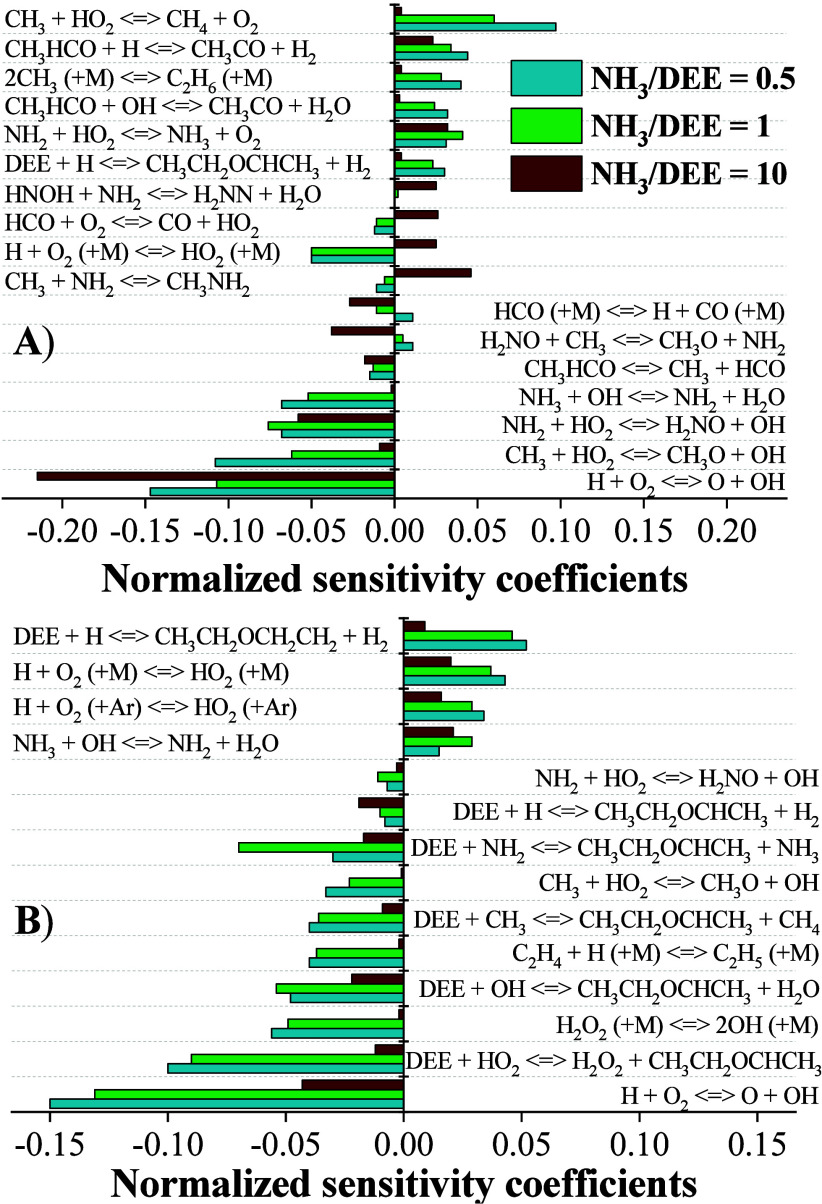
Sensitivity analysis for (A) NH_3_ and (B) DEE at 10% conversion for different NH_3_/DEE ratios. In ascending order of λ sets 5, 8, and 11 in Table 1.

Alternatively, DEE also competes for key radicals involved in NH_3_ consumption. This is evident in Figure 6A, where NH_3_ consumption is inhibited, and calculations reveal that reactions such as CH_3_CHO + OH ⇌ CH_3_CO + H_2_O (r51) are responsible for this effect. This effect is further reinforced by the fact that the hydrogen abstraction priority of DEE also plays a crucial role in NH_3_ inhibition. All these reactions become even more significant as the NH_3_/DEE mixture ratio decreases. Additionally, the consumption of CH_3_ radicals through alternative pathways, also contributes to the inhibition of NH_3_ consumption, as observed in reactions such as CH_3_ + HO_2_ ⇌ CH_4_ + O_2_ (r25) and 2CH_3_ (+M) ⇌ C_2_H_6_ (r26).

In NO production (Figure 5D), a distinct change in behavior occurs for the different NH_3_/DEE ratios are considered. For NH_3_/DEE = 10, the onset of NO formation occurs 200 K later than for the other conditions. Conversely, NO formation for NH_3_/DEE ≤ 1 takes place at similar temperatures. However, oxidation at equivalent fuel concentrations (i.e., NH_3_/DEE ≈ 1) results in higher NO emissions. In the higher temperature regions, it is noted that, in all cases, the calculations overestimate NO production compared to experimental data. This behavior change is attributed to a change in the reactions favoring the production of NO.

For NH_3_/DEE = 10, at the beginning of the reaction, it is evident that NO formation seems to be primarily driven by nitrogen chemistry, with reactions such as HNO + H ⇌ NO + H_2_ (r30), HNO + O_2_ ⇌ HO_2_ + NO (r52), and NH + O_2_ ⇌ NO + OH (r53). However, the reaction C_2_H_4_ + HNO ⇌ C_2_H_5_ + NO (−r29) also provides a notable contribution to the presence of NO. (−r29) plays a key role in the early stages of NO production for all the NH_3_/DEE ratios studied. Subsequently, the dominant reaction in NO generation shifts to CH_3_ONO (+M) ⇌ CH_3_O + NO (+M) (r54), indicating that DEE-derived compounds influence NO production across the entire temperature range in which NO is formed. There is a change in behavior at high temperatures. While at low temperatures the formation of NO is governed by the nitrogen chemistry with no apparent influence of DEE presence, at high temperatures, compounds such as HCN, HCN + OH ⇌ CH_2_ + NO (r55), and H_2_CN, H_2_CN + OH ⇌ CH_3_ + NO (r56), contribute to NO formation. For fuel mixtures with NH_3_/DEE ≥ 1, it is observed that nitrogen chemistry becomes less relevant, with interactions involving (−r29) and C_2_H_5_ + NO_2_ ⇌ CH_3_CHOH + NO (−r57) becoming more meaningful. It is also worthwhile to mention that (r54), once again, becomes predominant at higher temperatures. The importance of (r54) is not novel, as this could be corroborated in previous studies.^14^

N_2_O also exhibits a different behavior with variations for different NH_3_/DEE ratios. Although N_2_O is predicted to be formed by the calculations, experimentally, it is only detected at low DEE concentrations (NH_3_/DEE = 10), with concentrations not exceeding 20 ppm. For NH_3_/DEE ≥ 1, the main N_2_O production reaction is NH + NO ⇌ N_2_O + H (r58). However, as the DEE concentration increases, the reaction NCO + NO ⇌ N_2_O + CO (r59) increases in relevance. Globally, the calculated N_2_O concentrations decrease with higher DEE content in the mixture under these conditions.

HCN has only been identified at NH_3_/DEE = 1. HCN is detected at low temperatures when NH_3_/DEE ≤ 1, and calculations predict the same behavior. Nevertheless, one of the most significant reactions remains HCNH + H ⇌ HCN + H_2_ (r33) across all cases. As the NH_3_/DEE ratio decreases, reactions HCNH + CH_3_ ⇌ HCN + CH_4_ (r60) and CH_2_ + NO ⇌ HCN + OH (r61) become more prominent in the initial stages of HCN generation.

Although most nitrogen compounds exhibit different behaviors with changes in the NH_3_/DEE ratio, N_2_ initial production (Figure 5F) shifts the temperature only 25 K. This behavior is also reflected in the calculations, where the reactions NH_2_ + NO ⇌ N_2_ + H_2_O (r33) and NNH ⇌ N_2_ + H (r34) are the dominant pathways for N_2_ formation in all cases studied.

Concerning O_2_ consumption (Figure 5C), complete O_2_ conversion occurs with a temperature difference of 250 K. This variation increases because O_2_ consumption is enhanced by reactions leading to the formation of CH_3_CHO and CH_2_O, i.e. CH_3_CHOH + O_2_ ⇌ CH_3_CHO + HO_2_ (r62) and CH_2_OH + O_2_ ⇌ CH_2_O + HO_2_ (r63). Meanwhile, HCO + O_2_ ⇌ CO + HO_2_ (r44) remains one of the most significant O_2_ ways of consumption in the early stages of the combustion. In contrast, for NH_3_/DEE = 10, the reaction H + O_2_ ⇌ O + OH (r21) becomes more relevant, which may be attributed to the reduced consumption of H radicals due to the lower hydrogen abstraction from DEE.

Regarding H_2_ (Figure 5G), it is observed that its concentration peak shifts to a lower temperature as the DEE contribution to the mixture decreases.^35^ The calculations for all conditions suggest that hydrogen abstraction with the radical H, DEE + H ⇌ CH_3_CH_2_OCHCH_3_ + H_2_ (r3), is the predominant reaction contributing to H_2_ production.

Concerning DEE, the dominance of DEE when NH_3_/DEE ≤ 1 is once again evident. This can be seen in Figure 5B, where a shift in DEE oxidation occurs from 975 K onward. The same trend is observed in the predominant consumption reactions (10% DEE consumption), where the hydrogen abstraction reaction in the second carbon position is the dominant one in all the cases studied, with the intervention of H radicals being the most significant, DEE + H ⇌ CH_3_CH_2_OCHCH_3_ + H_2_ (r3). However, when NH_3_/DEE ≤ 1, hydrogen abstraction on the first carbon also plays an important role, as the third consumption reaction, DEE + H ⇌ CH_3_CH_2_OCH_2_CH_2_ + H_2_ (r37) occurs. Competition of DEE with NH_3_ for OH radicals is also evident, with the hydrogen abstraction reaction involving the OH radical being the second most important DEE oxidation reaction, DEE + OH ⇌ CH_3_CH_2_OCHCH_3_ + H_2_O (r4). Finally, although DEE decomposition remains significant at high DEE concentrations, DEE (+M) ⇌ C_2_H_5_OH + C_2_H_4_ (+M) (r5), it is under conditions where carbon chemistry is not predominant (NH_3_/DEE = 10) that (r5) becomes the most important reaction for DEE consumption.

Figure 6B shows a DEE sensitivity analysis at the temperature at which 10% of DEE is consumed. The importance of the various radicals formed can be deduced, with the formation of OH radicals being crucial in mixtures containing significant amounts of DEE. This is supported by the relevance of reactions such as H + O_2_ ⇌ O + OH (r21), CH_3_ + HO_2_ ⇌ CH_3_O + OH (r23), and H_2_O_2_ (+M) ⇌ 2OH (+M) (r64). The hydrogen abstraction on the second carbon favors the oxidation of DEE in all the cases considered. Additionally, the promotion of hydrogen abstraction at other DEE positions is detrimental for DEE consumption, as indicated by DEE + H ⇌ CH_3_CH_2_OCH_2_CH_2_ + H_2_ (r37), which is the most inhibitory reaction for DEE oxidation.

Although intermediate DEE compounds such as C_2_H_5_OH (Figure 5J), C_2_H_6_ (Figure 5H) and CH_3_CHO (Figure 5M) do not show any apparent changes in the reactions that produce them, i.e., DEE (+M) ⇌ C_2_H_5_OH + C_2_H_4_ (+M) (r5), 2CH_3_ (+M) ⇌ C_2_H_6_ (+M) (r26), and CH_3_CH_2_OCHCH_3_ ⇌ C_2_H_5_ + CH_3_CHO (r40). However, C_2_H_5_OH presents the same discrepancy as before. Because of this, the same approach explained above was applied to one of the specific conditions (λ = 2; NH_3_/DEE = 1), resulting in a notable improvement. These figures are shown in the Supporting

Information, Figure S11.

Other compounds exhibit different behavior depending on the NH_3_/DEE mixture ratio. An example of this is C_2_H_4_ (Figure 5I), whose formation reaction is primarily C_2_H_5_ (+M) ⇌ C_2_H_4_ + H (+M) (−r7). However, at low DEE concentrations in the mixture (NH_3_/DEE = 10), DEE (+M) ⇌ C_2_H_5_OH + C_2_H_4_ (+M) (r5) plays a more significant role in the production of C_2_H_4_.

Another interesting compound to analyze is CH_4_. This is because, for high NH_3_/DEE ratios, reactions involving the methyl radical interacting with CH_3_CHO and CH_2_O, such as CH_3_CHO + CH_3_ ⇌ CH_3_CO + CH_4_ (r50) and CH_2_O + CH_3_ ⇌ HCO + CH_4_ (r65), are the main sources of CH_4_. However, as the DEE concentration increases, methyl radicals contribute importantly to the radical pool. Consequently, the hydrogen abstraction of DEE by CH_3_ also becomes more significant, with reactions such as DEE + CH_3_ ⇌ CH_3_CH_2_OCHCH_3_ + CH_4_ (r6) and DEE + CH_3_ ⇌ CH_3_CH_2_OCH_2_CH_2_ + CH_4_ (r49).

It should be noted that the production of CO (Figure 5K) and CO_2_ (Figure 5L) is shifted to higher temperatures as the NH_3_/DEE ratio increases. The onset of CO and CO_2_ formation occurs at temperature differences of 50 and 100 K, respectively. Simultaneously, the concentrations obtained under these conditions vary depending on the initial DEE concentration. For CO_2_, no significant variation in behavior has been reported in the calculations. In contrast, CO exhibits differences in its initial generation. When NH_3_/DEE = 10, CO production primarily occurs through HCO + O_2_ ⇌ CO + HO_2_ (r44) and HCO (+M) ⇌ H + CO (+M) (r66). As the NH_3_/DEE ratio increases, reaction (r64) becomes less significant, while the decomposition of CH_3_CO, CH_3_CO (+M) ⇌ CH_3_ + CO (+M) (r14), gains prominence. This trend continues until CH_3_CO decomposition becomes the dominant CO formation pathway.

### 4.4 Reaction Pathway for NH_3_ and DEE Conversion

Figure 7 shows the reaction pathway of NH_3_ during pyrolysis (Figure 7A) and under different oxygen excess ratio conditions (Figure 7B), obtained with the model. Initially, ammonia is oxidized through its interaction with the H radical, NH_3_ + H ⇌ NH_2_ + H_2_ (r16). The NH_2_ radical interacts in two distinct ways: it either reconverts to ammonia upon interaction with C_2_H_4_, C_2_H_4_ + NH_2_ ⇌ C_2_H_3_ + NH_3_ (r28) or forms N_2_H_3_ when reacting with H radicals, 2NH_2_ ⇌ N_2_H_3_ + H (r67). This N_2_H_3_ is converted into tHNNH (r1), which interacts differently depending on pressure conditions, either through H_2_NN or CHNNH (r2 and r3 reaction pathway, respectively). Although both reaction paths diverge when tHNNH is formed, the final compound produced is the same in both cases: N_2_.

$$\begin{array}{l} r1.\;{\mathrm{NH}}_{3}\to {\mathrm{NH}}_{2}\to N_{2}H_{3}\to \mathrm{tHNNH}\\ r2.\;\mathrm{tHNNH}\to H_{2}\mathrm{NN}\to N_{2}\\ r3.\;\mathrm{tHNNH}\to \mathrm{cHNNh}\to \mathrm{NNH}\to N_{2} \end{array}$$

The reaction pathway for NH_3_ is significantly different when O_2_ is added to the mixture. Although NH_3_ initially oxidizes to the NH_2_ radical, the medium in which this takes place is different, as H radicals are no longer significant; instead, the system becomes more sensitive to OH and O radicals. Subsequently, the NH_2_ radical transforms into H_2_NO through its interaction with the HO_2_ radical, NH_2_ + HO_2_ ⇌ H_2_NO + OH (r22). This reaction pathway is defined as r4. Pathways described above are also present for the different stoichiometries λ studied (Figure 7B).

$$r4.,{\mathrm{NH}}_{3}\to {\mathrm{NH}}_{2}\to H_{2}\mathrm{NO}$$

Other reaction paths of NH_3_ differentiate between fuel-rich and other conditions. Under fuel-rich conditions, the NH_2_ radical couples with a methyl radical, leading to the reaction NH_2_ + CH_3_ (+M) ⇌ CH_3_NH_2_ (+M) (r68). It is remarkable the formation of this species which indicates the relevance of intermediate C–N species, as has been reported in studies dealing with methylamine conversion.^64,65^ This compound, through interaction with H and OH radicals, undergoes further decomposition, resulting in CH_2_NH. This defines the reaction path r5.

$$r5.,{\mathrm{CH}}_{3}{\mathrm{NH}}_{2}\to {\mathrm{CH}}_{2}{\mathrm{NH}}_{2}\to {\mathrm{CH}}_{2}\mathrm{NH}$$

The reaction of CH_2_NH with H and OH radicals produces HCNH. Consequently, a series of reactions are triggered, leading to the production of NH from HCNH, i.e., reaction paths r6 and r7.

$$\begin{array}{l} r6.,\mathrm{HCNH}\to \mathrm{HCN}\to \mathrm{NH}\\ r7.,\mathrm{HCNH}\to \mathrm{HNCO}\to \mathrm{NH} \end{array}$$

However, this is not the only sequence observed, as HCN and HNCO follow a different reaction pathway corresponding to the production and consumption of NCO. For NCO production, O_2_ is crucial, as its formation arises from HCN + O ⇌ NCO + H (r69) and CN + O_2_ ⇌ NCO + O (r70). In contrast, NCO consumption involves both nitrogenous compounds, with HNCO + NH_2_ ⇌ NH_3_ + NCO (r71), and short-chain hydrocarbons, such as C_2_H_6_ + NCO ⇌ C_2_H_5_ + HNCO (r72). All this results in reaction pathway (r8), also producing NH radicals.

r8.⁣HCN/HNCO→NCO→HNCO→NH

The NH radicals produced from reaction pathways r6, r7, and r8 are fundamental, as the majority of the emissions species of interest are derived from this intermediate compound. NH passes through two different reaction pathways, each involving a pollutant of interest. In r9, N_2_O is generated from NH + NO ⇌ N_2_O + H (r58), and is subsequently oxidized to N_2_ with the assistance of the H radical, N_2_O + H ⇌ N_2_ + OH (r73), and carbon monoxide, N_2_O + CO ⇌ N_2_ + CO_2_ (r74). In contrast, in r10, HNO is formed from NH, NH + O_2_ ⇌ HNO + O (r75). HNO is one of the key compounds in the production of NO, through reactions such as C_2_H_4_ + HNO ⇌ C_2_H_5_ + NO (−r29) and HNO + CH_3_ ⇌ NO + CH_4_ (r76). This leads to the conclusion that DEE-derived carbon compounds play a key role in the production of NO in these particular cases.

$$\begin{array}{l} r9.,\mathrm{NH}\to N_{2}O\to N_{2}\\ r10.,\mathrm{NH}\to \mathrm{HNO}\to \mathrm{NO}\to N_{2} \end{array}$$

For cases where sufficient O_2_ is available to oxidize the entire fuel mixture, the reaction pathways are less complex, consisting of only three pathways. This change occurs via the H_2_NO structural isomerization in HNOH. The thermal decomposition of HNOH leads to the r11 reaction pathway.

$$r11.,\mathrm{NHOH}\to H_{2}\mathrm{NN}\to \mathrm{tHNNH}\to N_{2}H_{3}$$

However, interactions with the HO_2_ radical become more relevant, leading to the formation of NH_2_OH or HNO. NH_2_OH follows a short path, decomposing back into NH_2_. In comparison, HNO exhibits the same behavior as described in r10.

**Figure 7:**
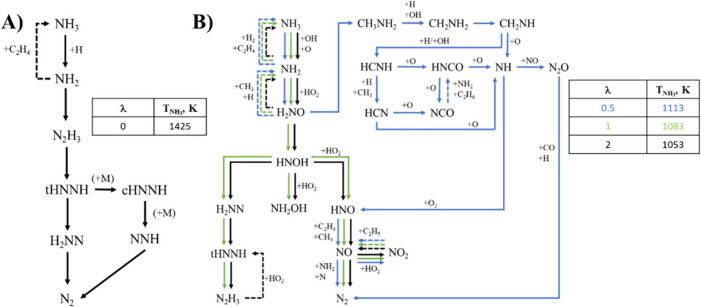
Reaction pathways of (A) NH_3_ pyrolysis conditions and for (B) different λ.

Different NH_3_/DEE ratios have been examined, with the full reaction pathway diagram provided in Figure S12 of the Supporting Information. The most significant changes focus on the pathway through which N_2_ is produced. While a higher DEE concentration (NH_3_/DEE = 0.5) does not reveal a new significant pathway for N_2_ production, at NH_3_/DEE ≥ 1 two distinct pathways appear. In both cases under study, a substantial portion of the N_2_ produced results from the NO reduction. Conversely, when there is a lower concentration of DEE in the mixture, it is observed that r12 gains relevance. All of the above points to a clear predominance of DEE over NH_3_, with more NH_3_ reaction pathways in cases where the DEE concentration does not exceed that of NH_3_.

$$r12.,\mathrm{tHNNH}\to \mathrm{NNH}\to N_{2}$$

For DEE, thermal decomposition is the most important process in the absence of O_2_, i.e. DEE (+M) ⇌ C_2_H_5_OH + C_2_H_4_ (+M) (r5). This reaction produces C_2_H_5_OH and C_2_H_4_, two of the most important intermediate compounds in the oxidation of DEE. The ethanol formed leads to an increase in C_2_H_4_ production, as specified in r13. The formation of C_2_H_4_ accounts for the subsequent appearance of C_2_H_2_ through C_2_H_3_ + H (+M) ⇌ C_2_H_2_ + H_2_ (+M) (r77). Acetylene generates other compounds, which are marginal in the experiments of the present work. The entire reaction pathway is represented in r14. Finally, C_2_H_4_ also gives rise to ethane, C_2_H_4_ + CH_3_ ⇌ C_2_H_6_ + CH (r78), although this pathway (r15) is less significant.

$$\begin{array}{l} r13.\;\mathrm{DEE}\to C_{2}H_{5}\mathrm{OH}\to {\mathrm{SC}}_{2}H_{4}\mathrm{OH}\to C_{2}H_{3}\mathrm{OH}\to C_{2}H_{4}\\ r14.\;\mathrm{DEE}\to C_{2}H_{4}\to C_{2}H_{3}\to C_{2}H_{2}\to C_{3}H_{4}\mathrm{-P}\to C_{3}H_{3}\\ r15.\;C_{2}H_{4}\to C_{2}H_{6}\to C_{2}H_{5} \end{array}$$

Although it is a less significant pathway in pyrolysis, it is important to mention the reaction pathway caused by the hydrogen abstraction arising in DEE from H radicals (r16), which forms CH_3_CH_2_OCHCH_3_. As a result, the radical undergoes complete decomposition via CH_3_CH_2_OCHCH_3_ ⇌ CH_3_CHO + C_2_H_5_ (r79). Acetaldehyde is also one of the most relevant intermediate compounds in the oxidation of DEE, from which the final CO and CO_2_ emissions are derived.

$$r16.\;\mathrm{DEE}\to {\mathrm{CH}}_{3}{\mathrm{CH}}_{2}{\mathrm{OCHCH}}_{3}\to {\mathrm{CH}}_{3}\mathrm{CHO}\to {\mathrm{CH}}_{3}\mathrm{CO}\to \mathrm{CO}\to {\mathrm{CO}}_{2}$$

Figure 8B shows the DEE reaction paths for NH_3_/DEE = 1 for different λ. In all cases, the predominant reaction pathway is the one indicated in r16, where hydrogen abstraction is produced by the H radical. This hydrogen abstraction also leads to the formation of C_2_H_5_, which decomposes to form C_2_H_4_. Ethylene is also produced through the thermal decomposition of DEE, DEE (+M) ⇌ C_2_H_5_OH + C_2_H_4_ (+M) (r5). From ethylene, another important source of CO and CO_2_ emissions is generated. The reaction pathway in question is represented in the reaction pathway r17.

$$r17.\;\mathrm{DEE}/C_{2}H_{5}\to C_{2}H_{4}\to C_{2}H_{3}\to {\mathrm{CH}}_{2}O\to \mathrm{HCO}\to \mathrm{CO}\to {\mathrm{CO}}_{2}$$

The only noticeable difference occurs in the case of fuel-rich conditions, where CO interacts with the NH_2_ radical, NH_2_ + CO ⇌ HNCO + H (r80). From CO via HNCO, two different product channels can take place: r18 and r19.

r18.⁣CO→HNCO→NCO

$$r19.,\mathrm{CO}\to \mathrm{HNCO}\to \mathrm{HNO}\to {\mathrm{CH}}_{4}$$

When HNCO reacts with CH_3_ radicals, it forms NCO (r18). This compound produces HNCO again through the reaction NH_3_ + NCO ⇌ HNCO + NH_2_ (−r81), thus favoring the oxidation of ammonia. If HNCO reacts with O_2_ (r19), HNCO + O_2_ ⇌ HNO + CO_2_ (r82), the production of HNO is favored. Consequently, this leads to a generation of more C_2_H_5_, with HNO + C_2_H_4_ ⇌ NO + C_2_H_5_ (−r83), and favors the appearance of methane, via HNO + CH_3_ ⇌ NO + CH_4_ (r76). These reactions also result in NO emissions, leading to the conclusion that these carbon compounds produced during the fuel-rich conditions of the NH_3_/DEE mixture contribute to the final NO emissions.

**Figure 8:**
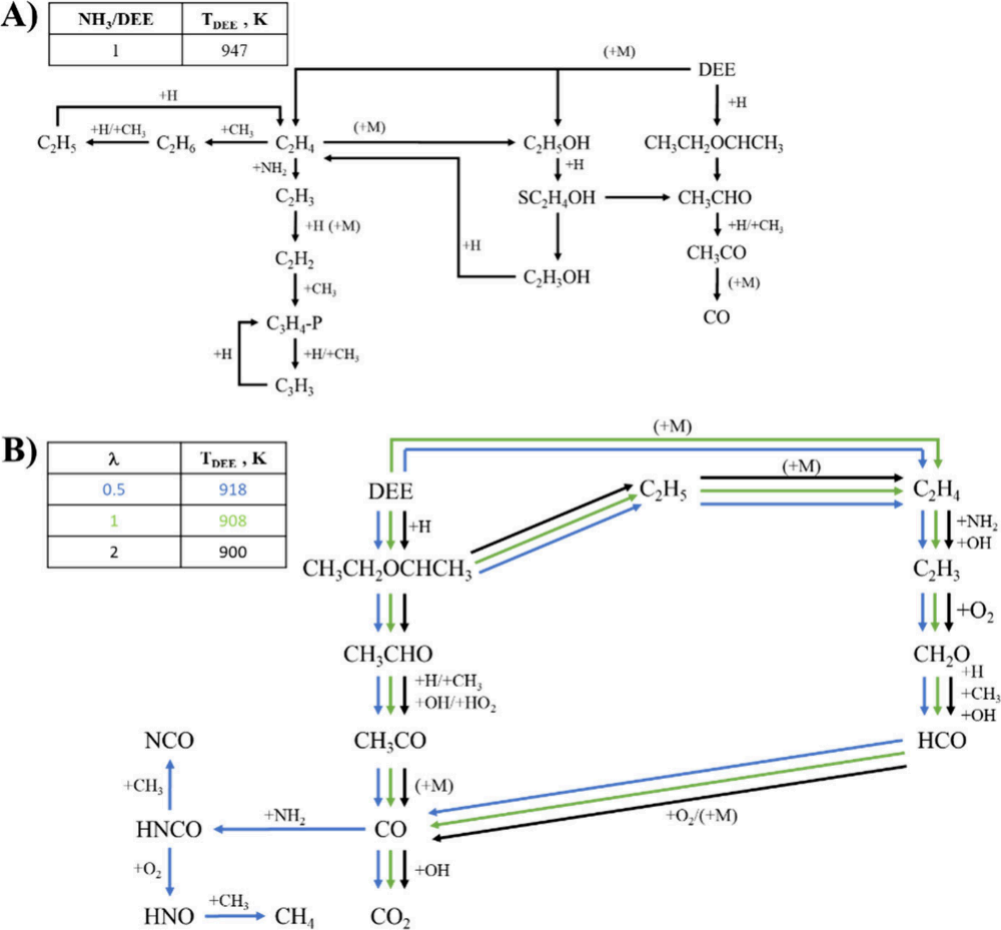
Reaction pathways of (A) DEE at pyrolysis and (B) DEE at different λ.

Finally, a study of the reaction pathways under fuel-lean conditions was conducted, varying the NH_3_/DEE mixture ratio. This reaction pathway is illustrated in Figure S12 of the Supporting Information. It is concluded that the reaction pathways under fuel-lean conditions do not vary significantly for different NH_3_/DEE ratios. This may be due to the predominance of DEE over NH_3_, which lowers the temperature at which DEE begins to oxidize, thus reducing competition for radicals. However, when the amount of DEE in the mixture is reduced (NH_3_/DEE = 10), the thermal decomposition of DEE becomes more significant again. This is because the temperature at which oxidation begins increases, leading to greater competition for radicals with NH_3_.

## 5 Conclusion

In this work, a comprehensive study of the conversion of the NH_3_/DEE fuel mixture has been conducted at atmospheric pressure in a flow reactor setup. For this purpose, the oxygen excess ratio (λ), NH_3_/DEE mixture ratio, and temperature have been systematically varied. In addition, a kinetic model has been updated and modified to achieve reliable representations of fuel mixture conversion studied in a plug flow reactor.

In pyrolysis, it is observed that DEE conversion is inhibited when mixed with NH_3_. This inhibition is due to competition for H radicals, which are essential for the initial oxidation of both fuels. The consumption of H radicals through secondary pathways leads to a reduction in DEE consumption. This is primarily attributed to the high importance of hydrogen abstraction, with H radicals playing the most significant role. In the absence of H radicals, the thermal decomposition of DEE becomes the dominant reaction. In contrast to DEE, NH_3_ does not exhibit high reactivity under pyrolysis conditions.

Regarding the oxygen excess ratio, the oxidation of both fuels occurs at lower temperatures as λ increases. This temperature difference becomes more pronounced at higher λ values, particularly in NH_3_ when λ < 1. The contribution of the radical pool is crucial in NH_3_ oxidation, with OH and O radicals being the most relevant. However, interactions with DEE derivatives have also been observed, and have been seen to promote NH_3_ oxidation. Additionally, fuel-lean conditions lead to higher NO emissions.

DEE is not strongly influenced by λ, as its reaction temperature range remains narrow. However, an increased O_2_ concentration promotes DEE oxidation at lower temperatures. OH radicals play a significant role under fuel-lean conditions, although hydrogen abstraction by H radicals remains the primary mechanism for DEE consumption. If H radicals are consumed through secondary pathways or extracted from areas outside the primary reaction zone, the oxidation of both fuels is hampered.

Concerning the NH_3_/DEE mixture ratio, the addition of DEE significantly alters ammonia oxidation. This effect is particularly evident in mixtures with higher DEE dilution (NH_3_/DEE = 10). Although OH and H radicals are the main contributors to the oxidation of the fuel mixture, an increased DEE ratio leads to a rise in the CH_3_ radicals, which also promotes fuel consumption.

The initial NO formation pathways are strongly influenced by the fuel mixture ratio. At higher NH_3_ concentrations, NO emissions primarily originate from nitrogen chemistry, whereas in NH_3_ diluted conditions, the opposite trend is observed. In general, NO emissions increase with the increasing reactivity of the mixture.

Finally, a kinetic model has been developed, and it provides reasonably accurate predictions under most conditions. However, further refinement is required, particularly for cases where DEE is more diluted, as well as for compounds such as N_2_O and C_2_H_5_OH, where predictive accuracy still has room for improvement.

## Supplementary Material



## Notes

Additional details regarding temperature profile and experimental setup (Figures S1–S3), nitrogen and carbon mass balances (Figure S4), calculations made with the original and updated mechanisms considered in the present work (Figure S5), sensitivity analysis under pyrolysis conditions (Figure S6), photo of the pyrolytic carbon found in the reactor under pyrolysis conditions (Figure S7), sensitivity analysis of NO under stoichiometric conditions at elevated temperatures (Figure S8), results obtained with another NH_3_/DEE ratio (Figures S9 and S10), results of relevant compounds when varying the reaction rate of DEE (+M) ⇌ C_2_H_5_OH + C_2_H_4_ (+M) (Figure S11), and reaction pathway diagrams of NH_3_ and DEE for different NH_3_/DEE ratios (Figure S12) (PDF)
