# Supplementary material for: Flow Reactor Study of NH3/DEE Oxidation Chemistry
Source: Energy Fuels. 2025 Aug 18;39(34):16407–21. doi: 10.1021/acs.energyfuels.5c01874 (PMC12400427; doi:10.1021/acs.energyfuels.5c01874)
Supplement: Supplementary file 1 [file ef5c01874_si_001.pdf]

A flow reactor study of NH_3/DEE oxidation chemistry

A. Ruiz-Gutiérrez, A. Bello-Gallego, M. U. Alzueta*

Aragón Institute of Engineering Research (I3A), Department of Chemical and Environmental Engineering, University of Zaragoza, 50018 Zaragoza, Spain

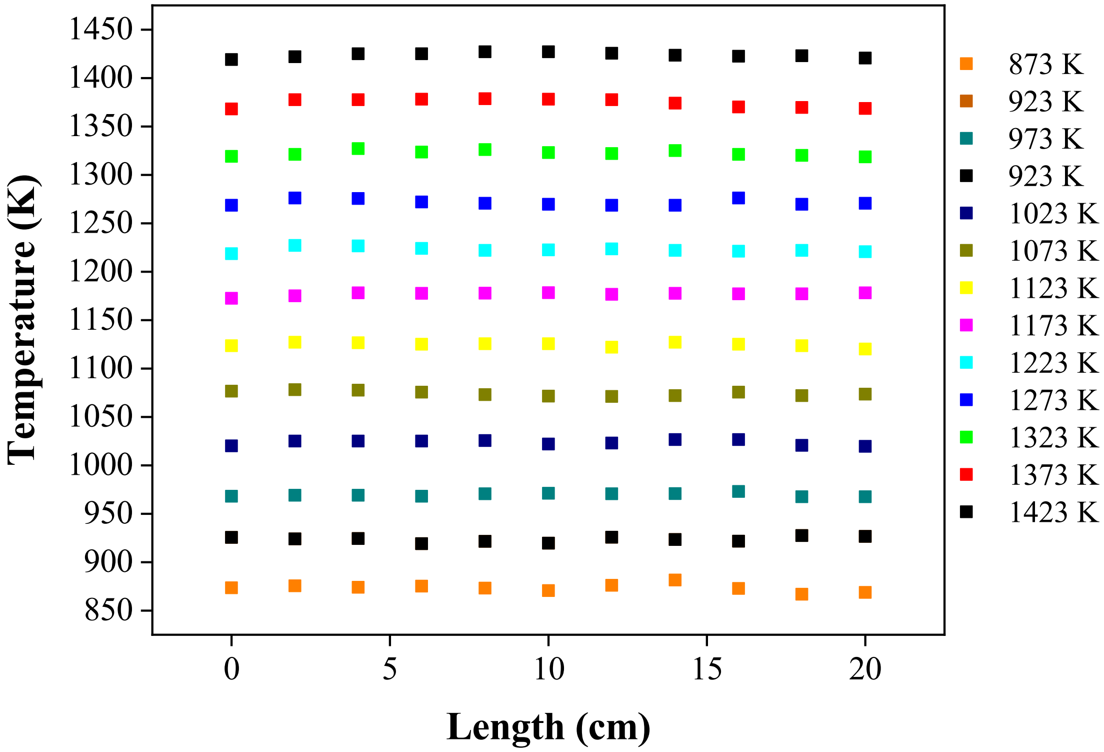

Figure S1. Flow reactor temperature profile.

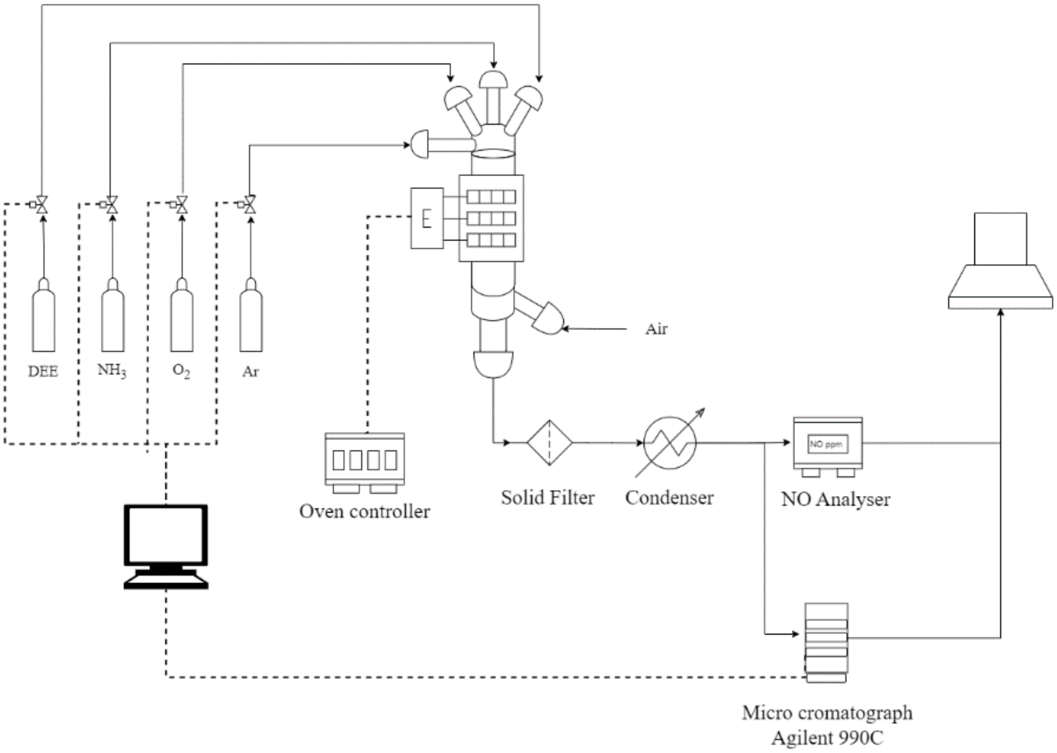

Figure S2. Experimental setup.

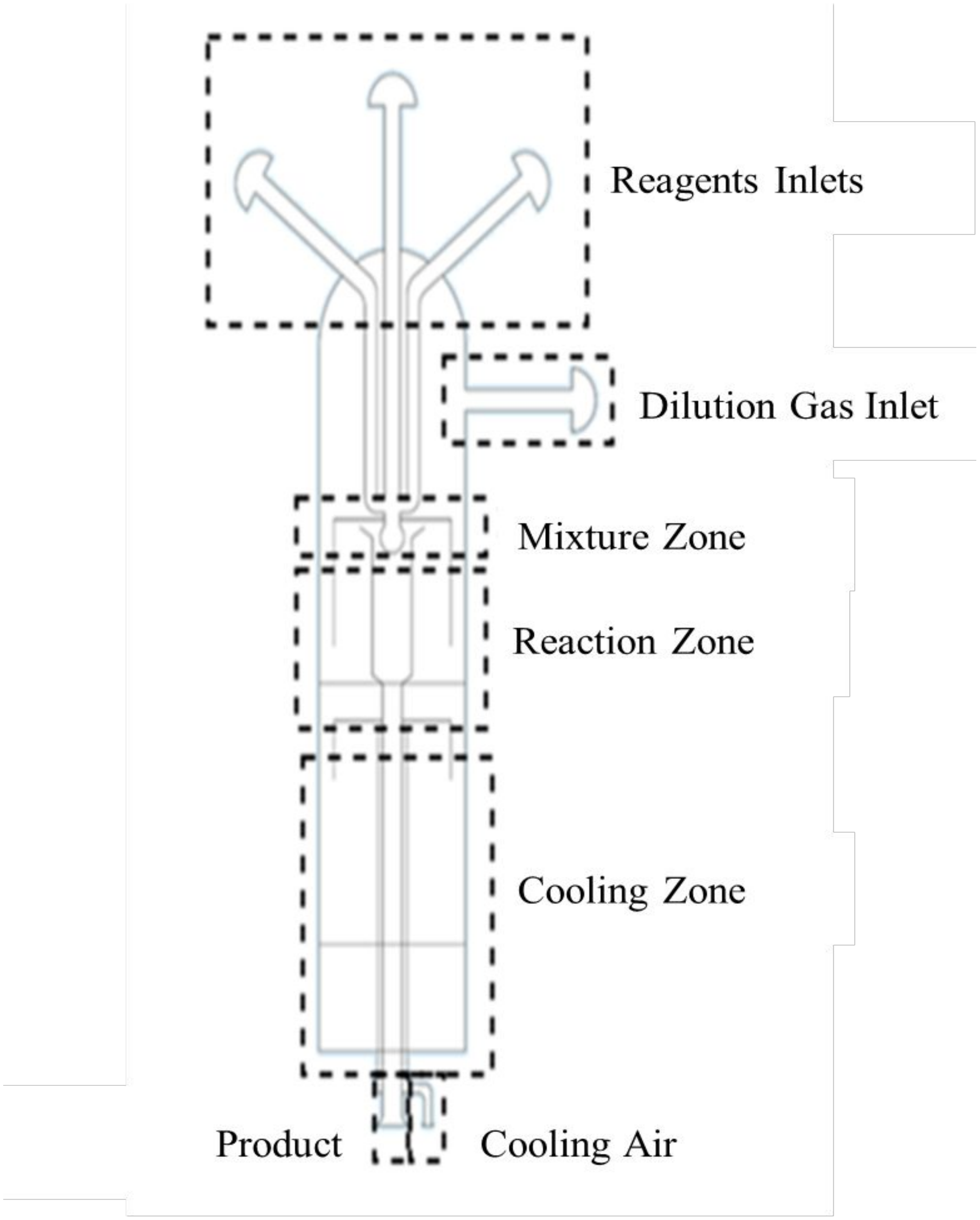

Figure S3. Flow reactor.

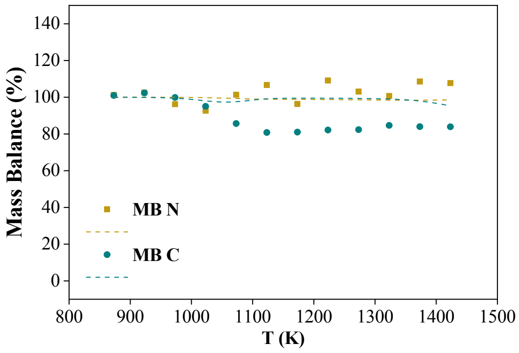

Fig S4.1. SET 1

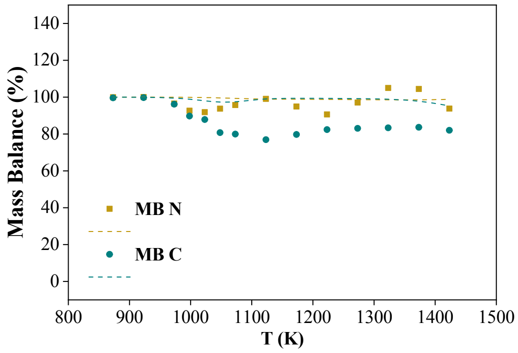

Fig S4.2. SET 2

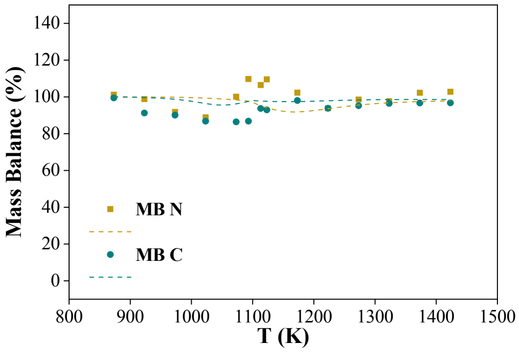

Fig S4.3. SET 3

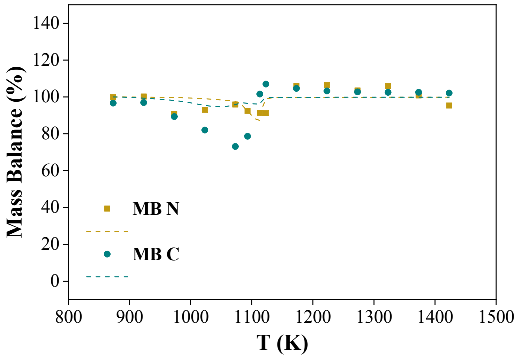

Fig S4.4. SET 4

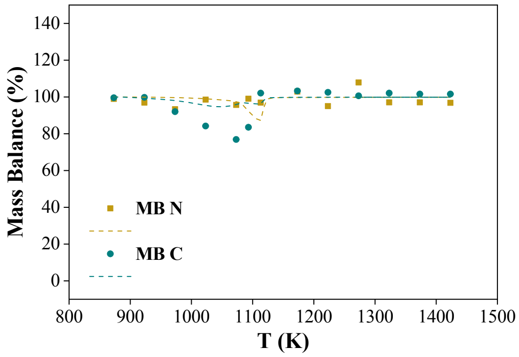

Fig S4.5. SET 4R

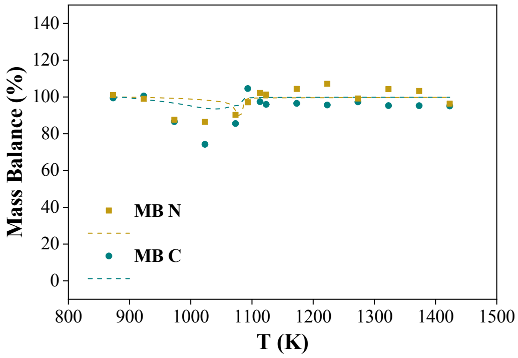

Fig S4.6. SET 5

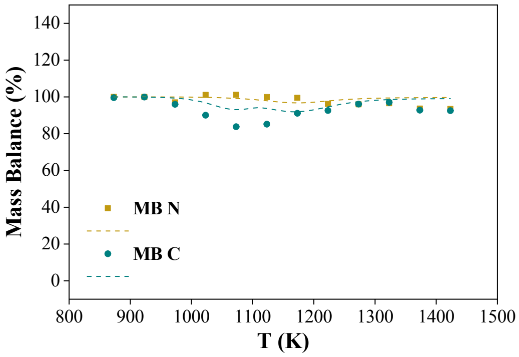

Fig S4.7. SET 6

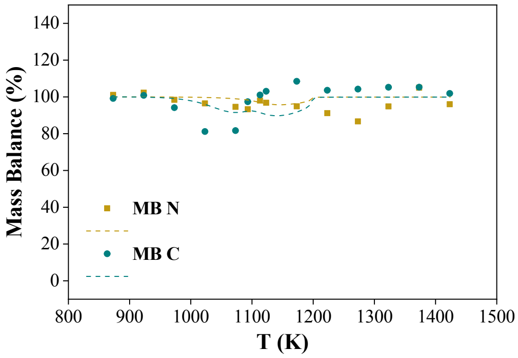

Fig S4.8. SET 7

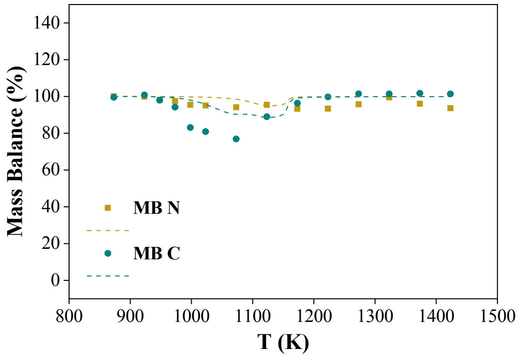

Fig S4.9. SET 8

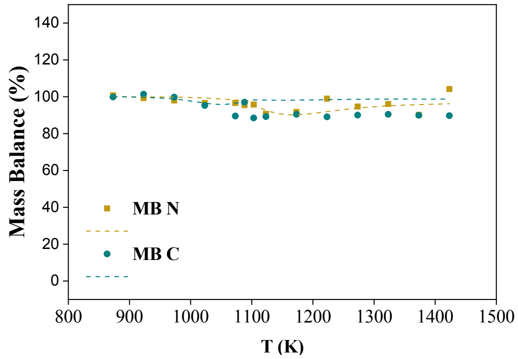

Fig S4.10. SET 9

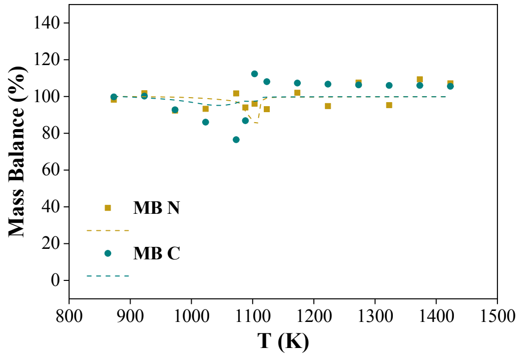

Fig S4.11. SET 10

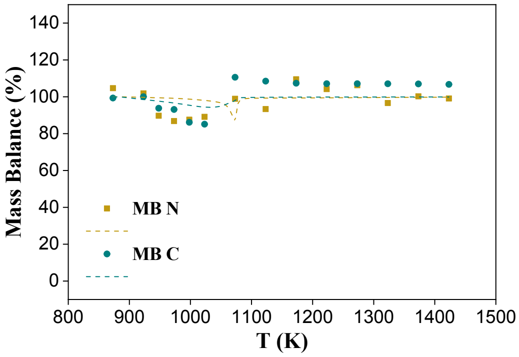

Fig S4.11. SET 11

Figure S4. N and C mass Balances for all experimental sets.

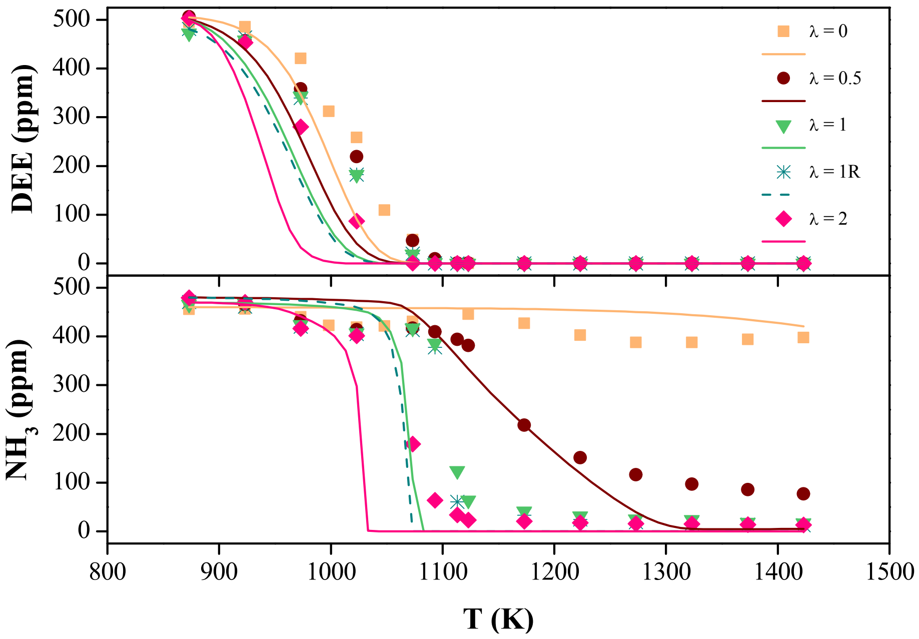

Figure S5.1. NH_3 and DEE species profile using the original mechanism.

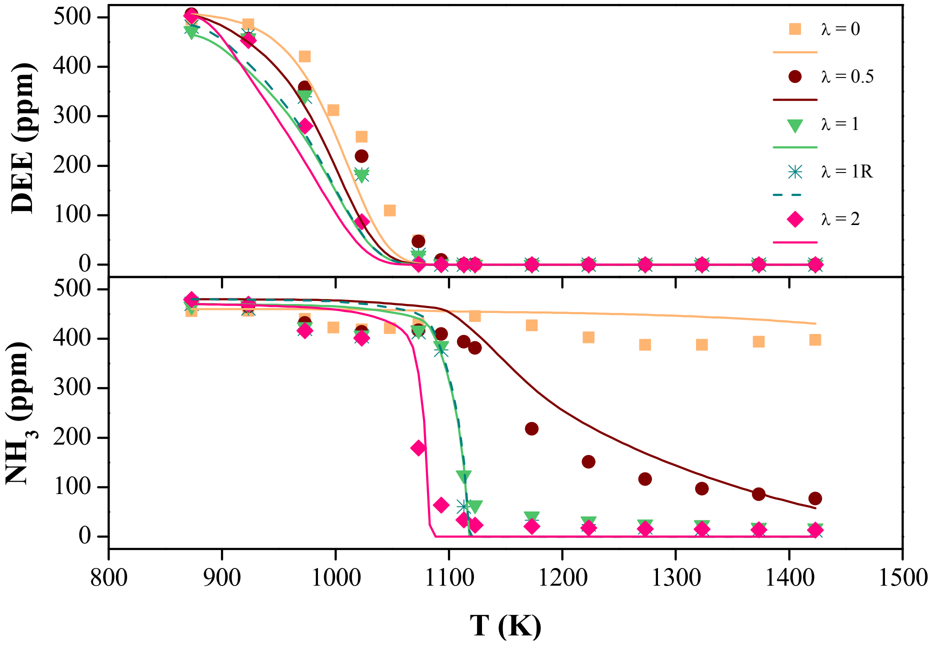

Figure 5.2. NH_3 and DEE species profile using the updated mechanism .

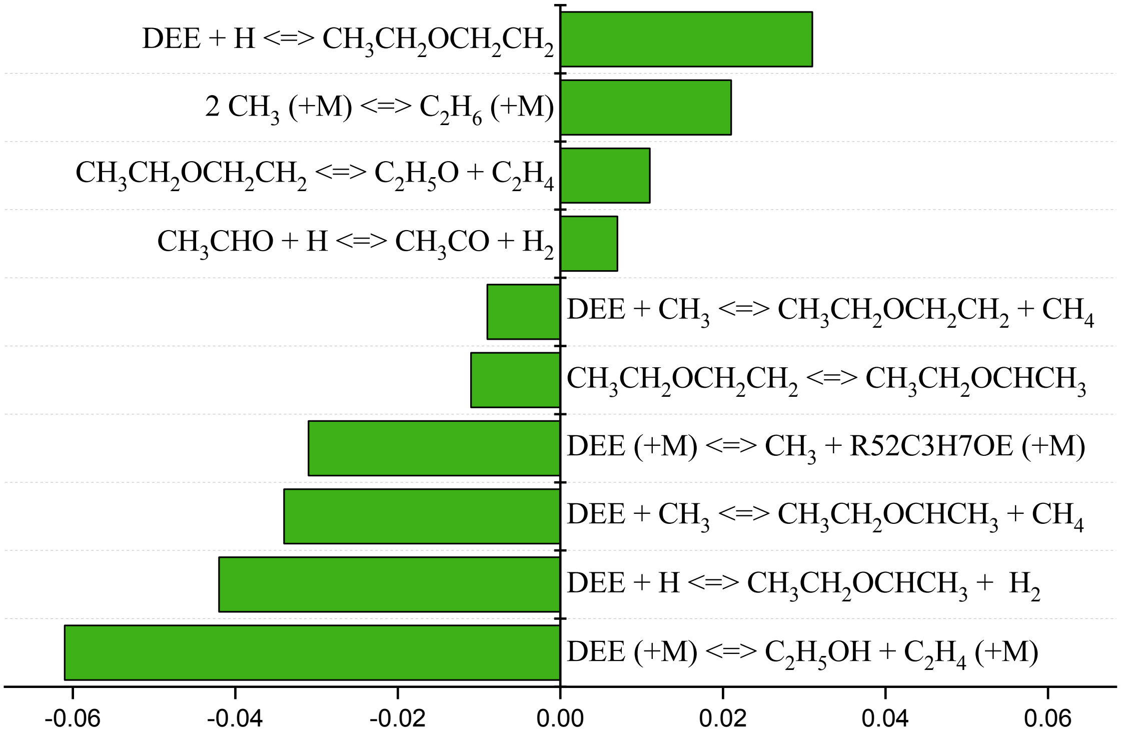

Figure S6.1. DEE sensitivity analysis at 948 K under pyrolysis conditions and NH_3/DEE = 1.

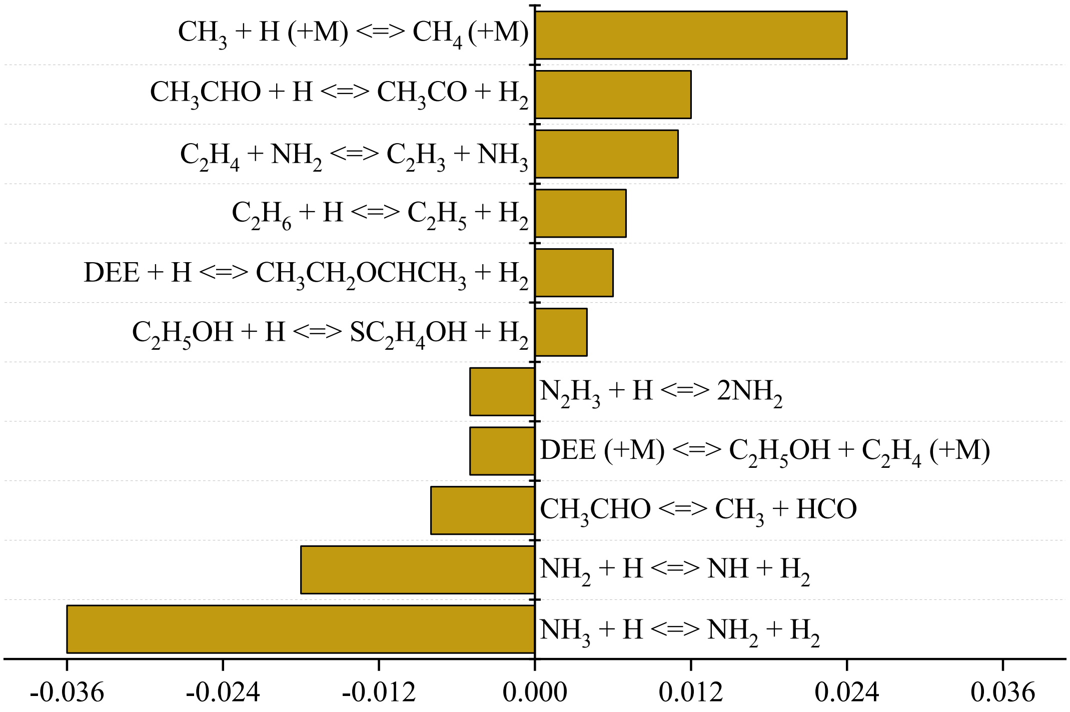

Figure S6.2. NH_3 sensitivity analysis at 1425 K under pyrolysis conditions and NH_3/DEE = 1.

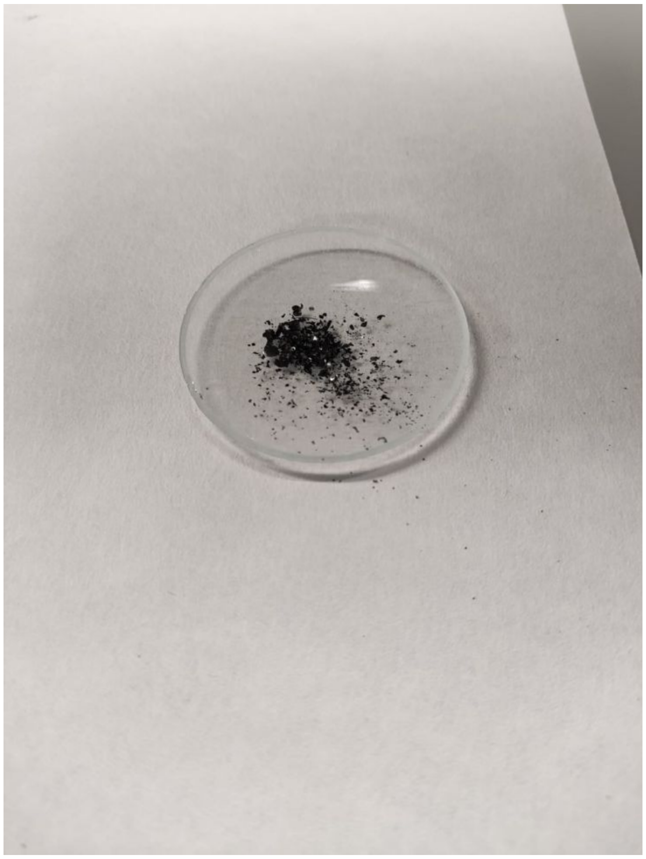

Figure S7. Pyrolytic carbon found in the reactor walls at the end of pyrolysis experiments.

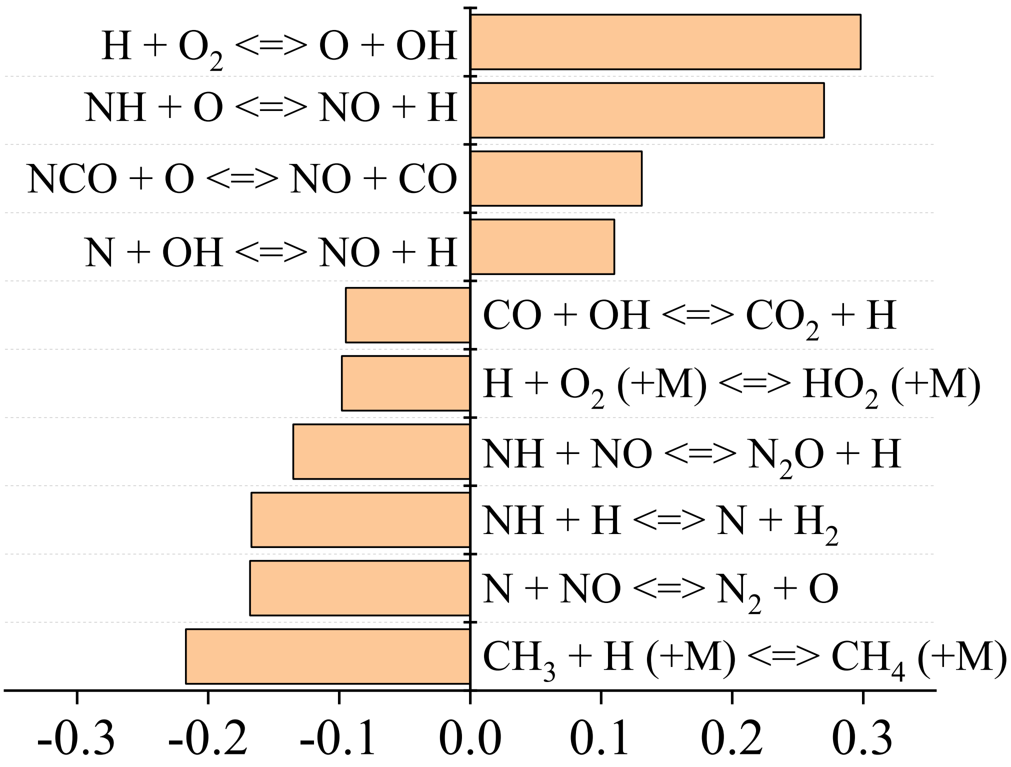

Figure S8. NO sensitivity analysis at 1250 K under stoichiometric conditions and NH_3/DEE = 1.

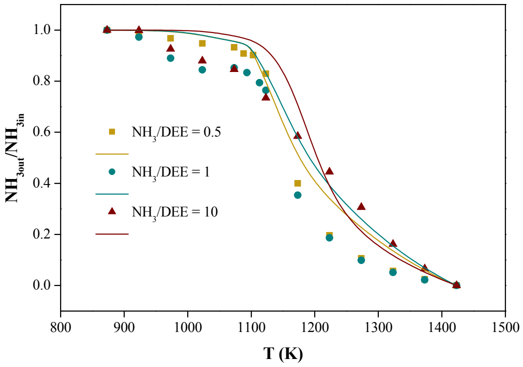

Fig S9.1. NH_3

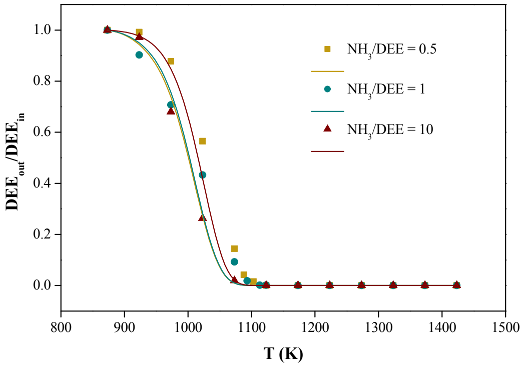

Fig S9.2. DEE

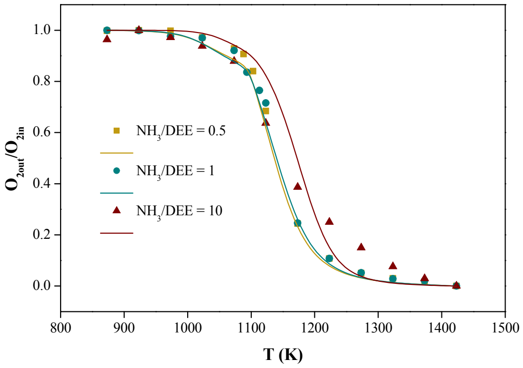

Fig S9.3. O_2

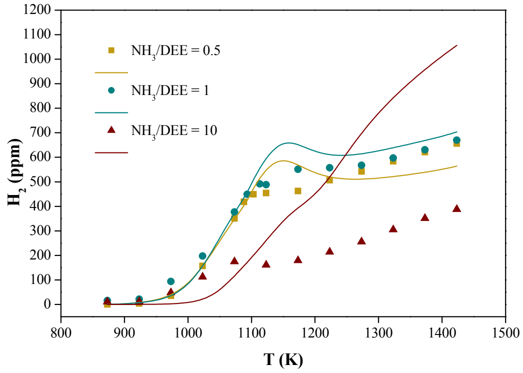

Fig S9.4. H_2

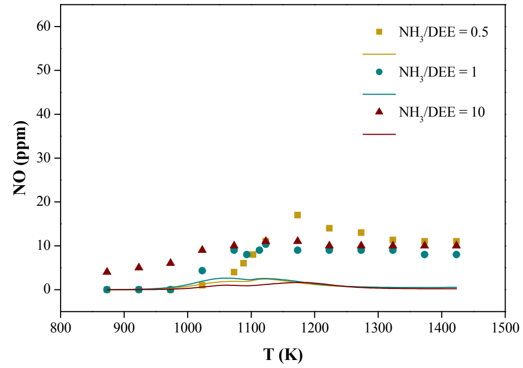

Fig S9.5. NO

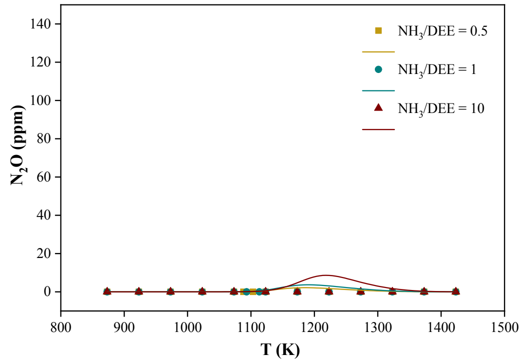

Fig S9.6. N_2O

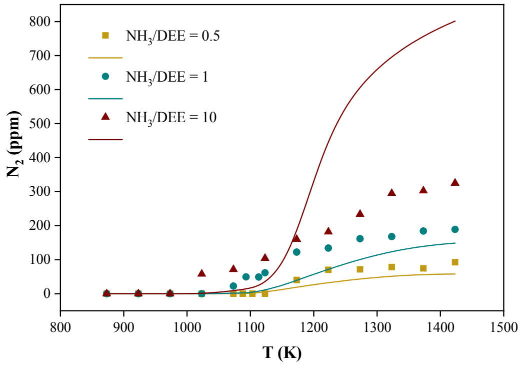

Fig S9.7. N_2

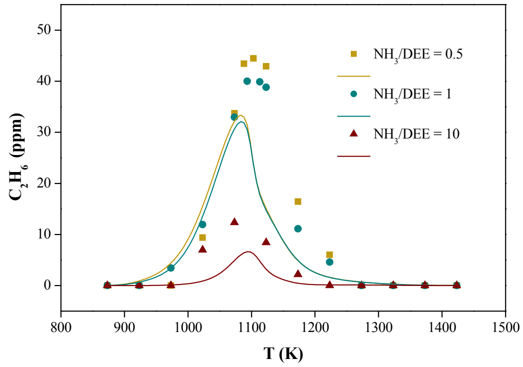

Fig S9.8. C_2H_6

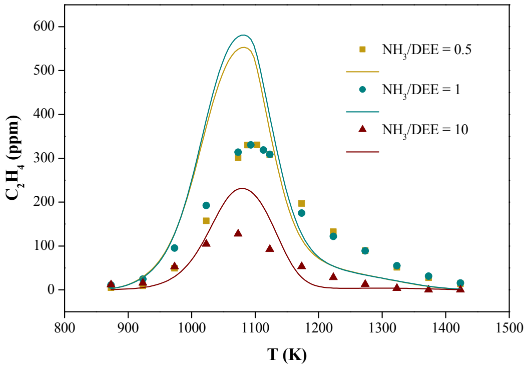

Fig S9.9. C_2H_4

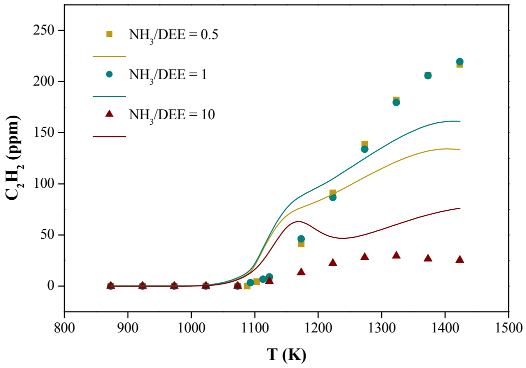

Fig S9.10. C_2H_2

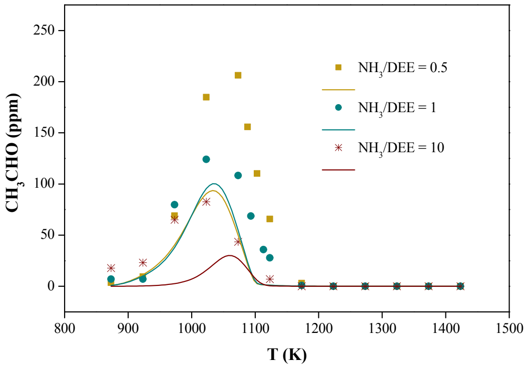

Fig S9.11. CH_3CHO

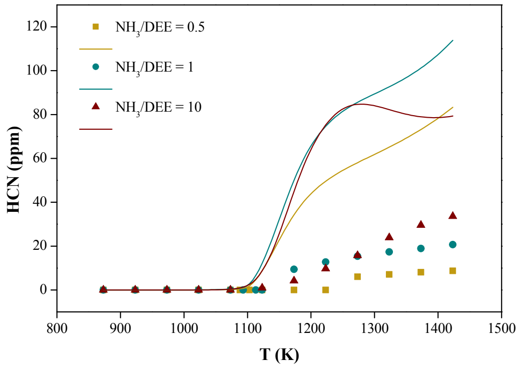

Fig S9.12. HCN

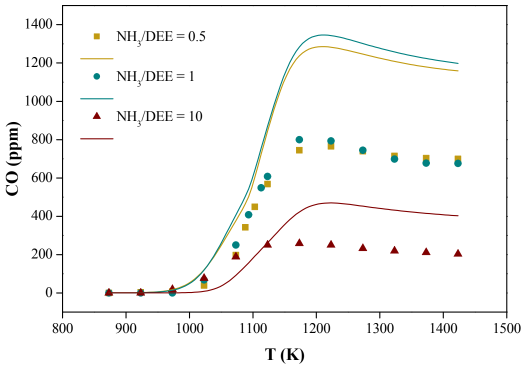

Fig S9.13. CO

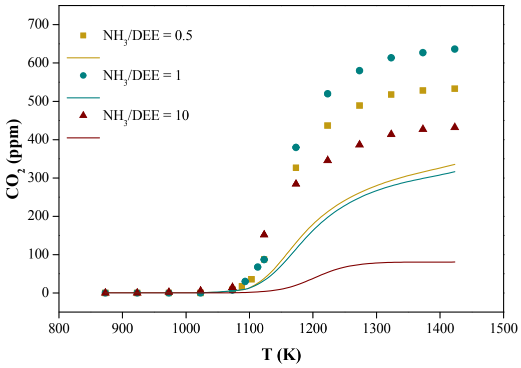

Fig S9.14. CO_2

Figure S9. Concentration of the different species quantified for λ = 0.5 and different NH_3/DEE ratios (Sets 3, 6 and 9 of Table 1).

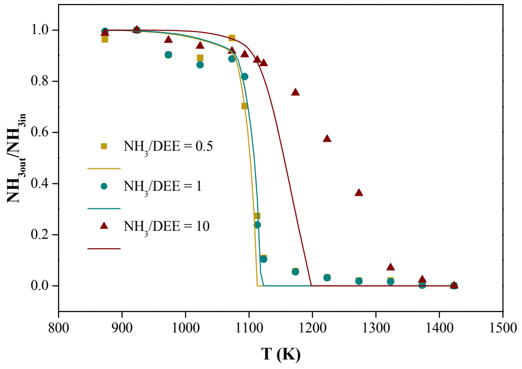

Fig S10.1. NH_3

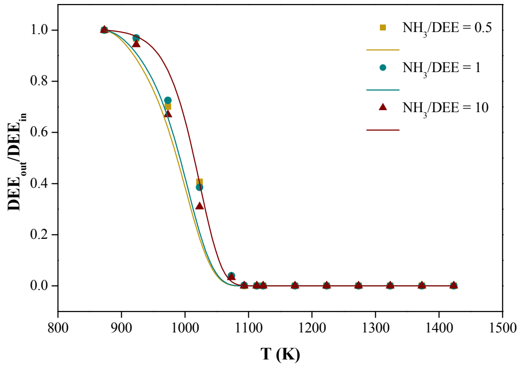

Fig S10.2. DEE

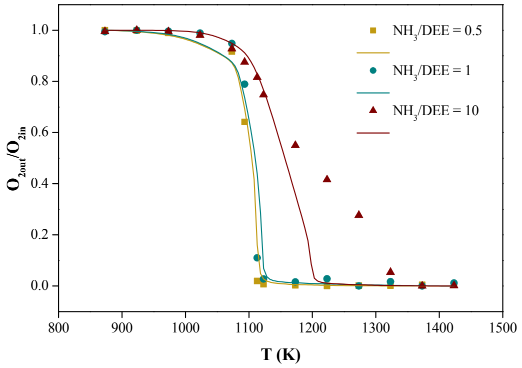

Fig S10.3. O_2

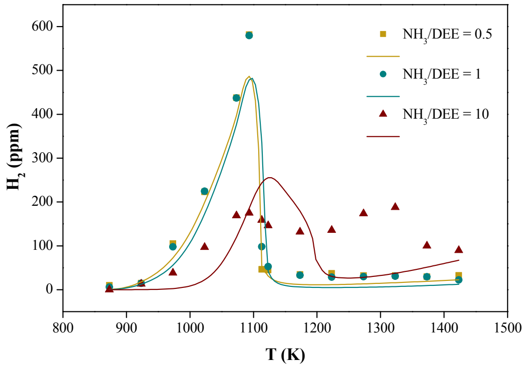

Fig S10.4. H_2

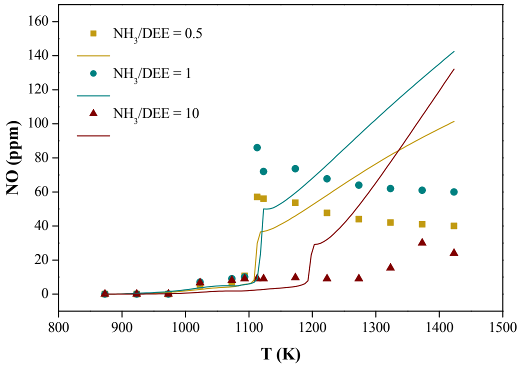

Fig S10.5. NO

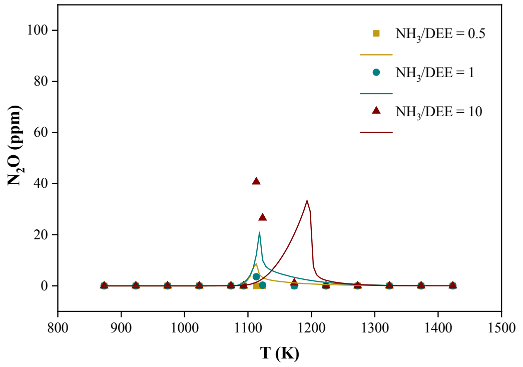

Fig S10.6. N_2O

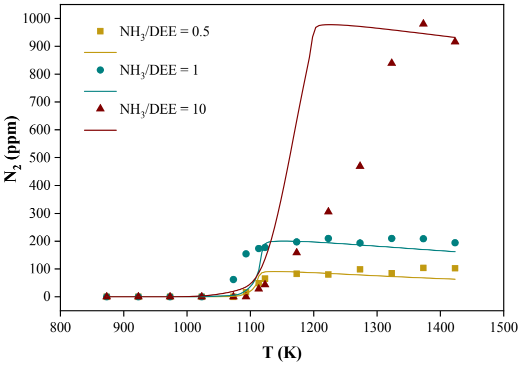

Fig S10.7. N_2

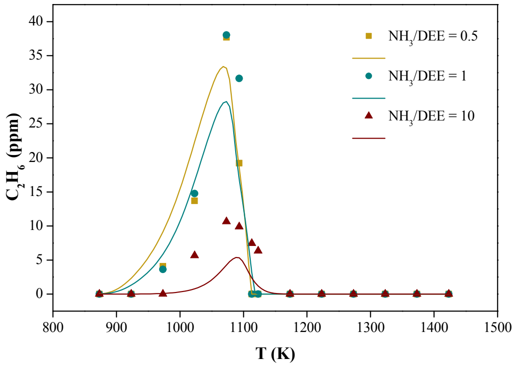

Fig S10.8. C_2H_6

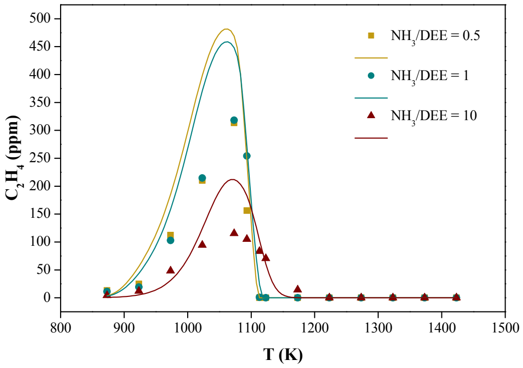

Fig S10.9. C_2H_4

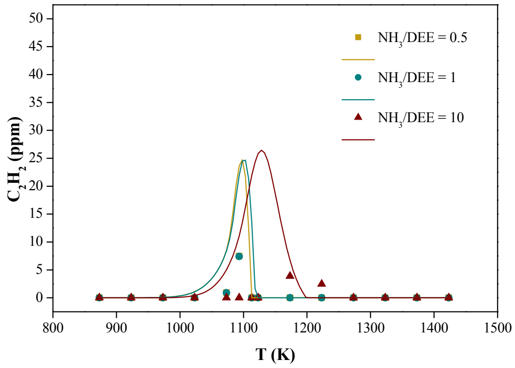

Fig S10.10. C_2H_2

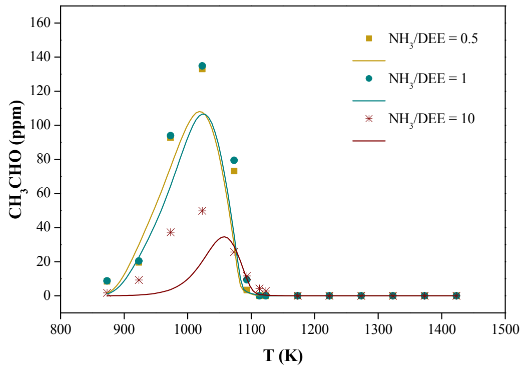

Fig S10.11. CH_3CHO

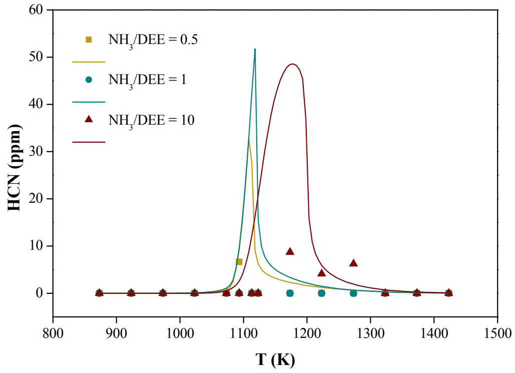

Fig S10.12. HCN

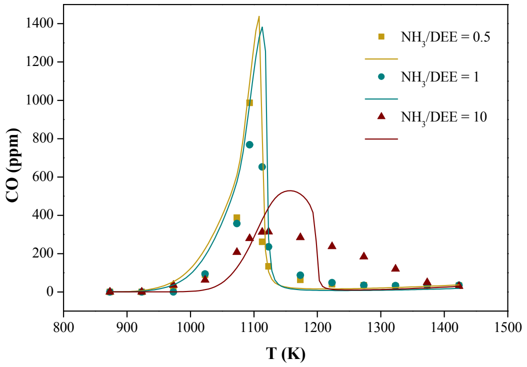

Fig S10.13. CO

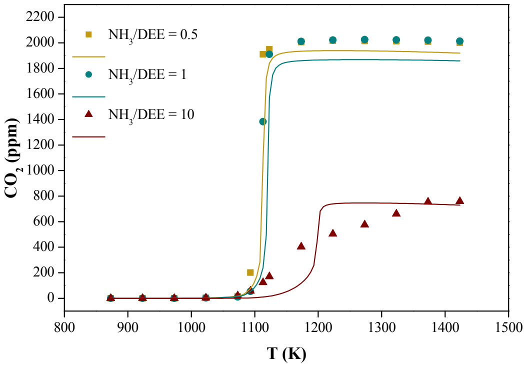

Fig S10.14. CO_2

Figure S10. Concentration of the different species quantified for $\lambda = 1$ and different NH_3/DEE ratios (Sets 4, 7 and 10 of Table 1).

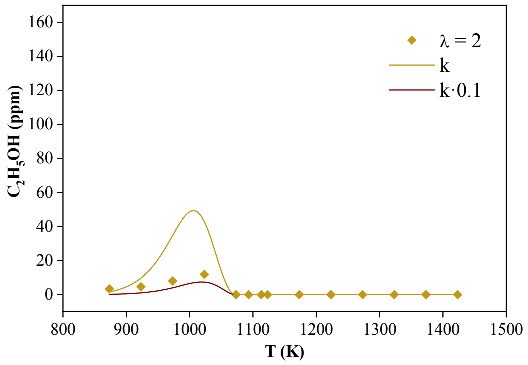

Figure 11.1. C_2H_5OH

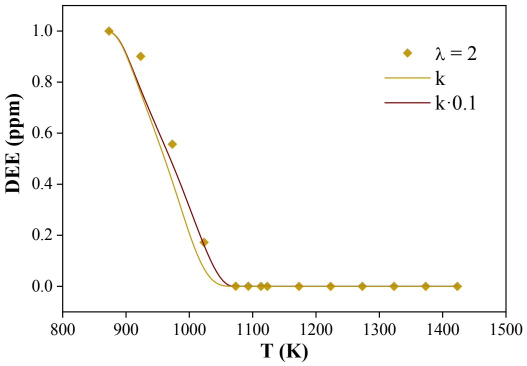

Figure 11.2. DEE

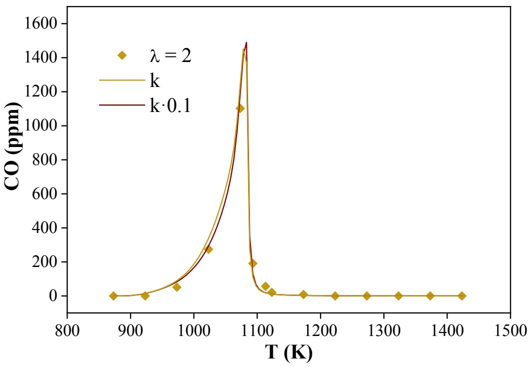

Figure 11.3. CO

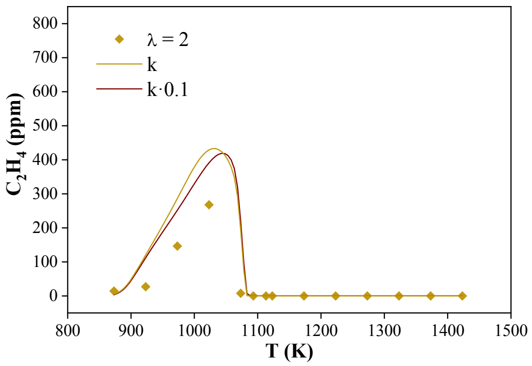

Figure 11.4. C_2H_4

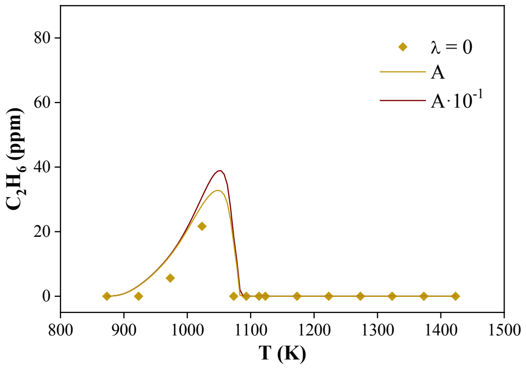

Figure 11.5. C_2H_6

Figure 11. Comparison of experimental results and calculations made with the Yasunaga et al. [60] rate for (r5), DEE (+M) ⇌ $C_2H_5OH + C_2H_4$ (+M), and the same rate divided by 10 for $\lambda = 2$ and NH_3/DEE = 1, Set *5* in Table 1.

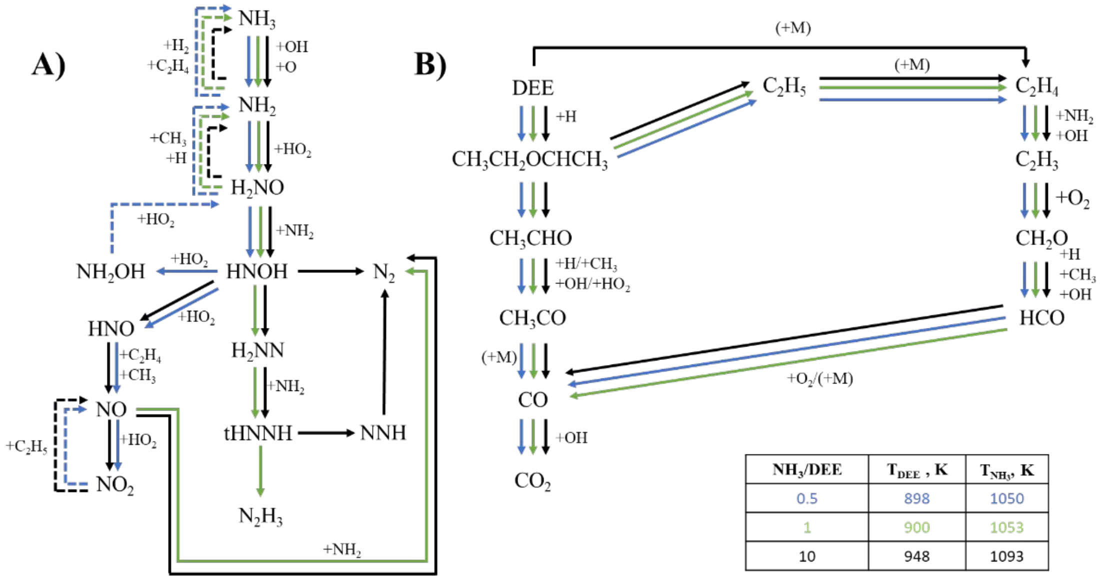

NH_3/DEE	T_{DEE} , K	T_{NH_3}, K
0.5	898	1050
1	900	1053
10	948	1093

Figure S12. Reaction pathways of NH_3 (A) and DEE (B) for different NH_3/DEE ratios.
